# Piperazine-derived lipid nanoparticles deliver mRNA to immune cells in vivo

**DOI:** 10.1038/s41467-022-32281-5

**Published:** 2022-08-15

**Authors:** Huanzhen Ni, Marine Z. C. Hatit, Kun Zhao, David Loughrey, Melissa P. Lokugamage, Hannah E. Peck, Ada Del Cid, Abinaya Muralidharan, YongTae Kim, Philip J. Santangelo, James E. Dahlman

**Affiliations:** 1 Wallace H. Coulter Department of Biomedical Engineering, Georgia Institute of Technology, Atlanta, GA USA; 2 Parker H. Petit Institute for Bioengineering and Bioscience, Georgia Institute of Technology, Atlanta, GA USA; 3 George W. Woodruff School of Mechanical Engineering, Georgia Institute of Technology, Atlanta, GA USA; 4 Institute for Electronics and Nanotechnology, Georgia Institute of Technology, Atlanta, GA USA; 5 Present Address: School of Pharmaceutical Sciences, Shandong University, Jinan, China

**Keywords:** Nucleic-acid therapeutics, Gene delivery, DNA, Nanoparticles, RNA nanotechnology

## Abstract

In humans, lipid nanoparticles (LNPs) have safely delivered therapeutic RNA to hepatocytes after systemic administration and to antigen-presenting cells after intramuscular injection. However, systemic RNA delivery to non-hepatocytes remains challenging, especially without targeting ligands such as antibodies, peptides, or aptamers. Here we report that piperazine-containing ionizable lipids (Pi-Lipids) preferentially deliver mRNA to immune cells in vivo without targeting ligands. After synthesizing and characterizing Pi-Lipids, we use high-throughput DNA barcoding to quantify how 65 chemically distinct LNPs functionally delivered mRNA (i.e., mRNA translated into functional, gene-editing protein) in 14 cell types directly in vivo. By analyzing the relationships between lipid structure and cellular targeting, we identify lipid traits that increase delivery in vivo. In addition, we characterize Pi-A10, an LNP that preferentially delivers mRNA to the liver and splenic immune cells at the clinically relevant dose of 0.3 mg/kg. These data demonstrate that high-throughput in vivo studies can identify nanoparticles with natural non-hepatocyte tropism and support the hypothesis that lipids with bioactive small-molecule motifs can deliver mRNA in vivo.

## Abstract (web summary)

Next-generation lipid nanoparticles that target non-hepatocytes could be important clinical tools. Using in vivo DNA barcoding, the authors identify piperazine-containing lipids deliver mRNA to immune cells without targeting ligands.

## Introduction

The Food and Drug Administration (FDA) approved its first lipid nanoparticle (LNP)-based siRNA drug to treat an inherited genetic disease in 2018^1^. Since then, systemically administered siRNA therapeutics using N-acetylgalactosamine^2^ have been approved to treat additional liver diseases^3–5^. Similarly, intramuscularly administered mRNA therapies have been FDA approved^6, 7^ to vaccinate against coronavirus disease in 2019. Unfortunately, there have also been clinical failures potentially driven by insufficient delivery^8, 9^. Taken together, the efficacy of approved RNA vaccines and liver therapies underscores the potential clinical impact of LNPs with tropism to new cell types.

Delivering RNA to non-hepatocytes^10, 11^ has remained challenging in large part due to the anatomy and physiology of the liver. Specifically, the hepatic sinusoids contain a discontinuous vasculature^12^ as well as slow blood flow^13^; both increase nanoparticle extravasation and subsequent interactions with hepatocytes. To target non-hepatocytes, scientists have used three approaches^14^. The first is to pre-treat animals with systems that overwhelm the liver^15^ or reduce drug activity^16^ in specific cell types, thereby shifting tropism. However, it remains unclear whether this multistep strategy has clinical relevance. In the second approach, an LNP with tropism to hepatocytes is retargeted with an active targeting ligand. For example, LNPs made with DLin-MC3-DMA^17^, an ionizable lipid that is FDA approved for hepatocyte siRNA delivery^18^, have been retargeted to immune cells using a lipid-bound antibody^19–22^. One potential limitation of this approach is that actively targeted nanoparticles containing RNA drugs have led to adverse events in clinical trials^23^; one important caveat is that these were not LNPs. In a third approach, scientists identify nanoparticles that interact with natural trafficking pathways, thereby leading to endogenous targeting^24^. Although these approaches have led to an FDA approval^18^ and promising phase 1 clinical data^25^, this approach also has a key limitation. After synthesizing a large, chemically diverse lipid library, scientists must evaluate how each nanoparticle delivers its payload into cells. Since injecting and sacrificing thousands of mice per library is unethical, this screening is performed in vitro (i.e., in cell culture). For example, across three representative papers^26–28^, labs tested 4,736 nanoparticles in vitro, using the data to select 14 nanoparticles for in vivo studies. However, this screening method is likely inefficient, given that in vitro nanoparticle delivery can be a poor predictor of in vivo nanoparticle delivery^29^.

One potential solution to this problem is to study many nanoparticles in a single animal, which has recently become feasible using DNA-barcoded nanoparticles^30, 31^. Here we use Fast Identification of Nanoparticle Delivery (FIND)^32^, a DNA barcode-based high-throughput LNP screening system, to characterize how 65 chemically distinct LNPs functionally deliver gene-editing mRNA to 14 cell types in vivo. FIND allowed us to test a chemical hypothesis directly in vivo: that medicinal chemistry scaffolds with known bioactivity could be incorporated into the ionizable lipids used in LNPs. We reasoned that these motifs could lead to distinctive in vivo activity, compared to canonical motifs used in many LNPs to date. We therefore focused on piperazine, a six-membered nitrogen-containing heterocycle, for several reasons. First, piperazine is commonly used in biologically active compounds^33^; 13 of the 200 best-selling small-molecule drugs in 2012 contained a piperazine ring^34^. Second, and relatedly, the piperazine ring has been recognized as the key structural motif in marketed drugs ranging from antidepressants^35, 36^ to antibiotics^37, 38^, demonstrating its utility in medicinal chemistry. Third, as a result of piperazine’s impact on small-molecule drugs, there are numerous piperazine-based chemistries^34^ that could be repurposed to synthesize diverse ionizable lipids.

## Results

**Fig. 1:**
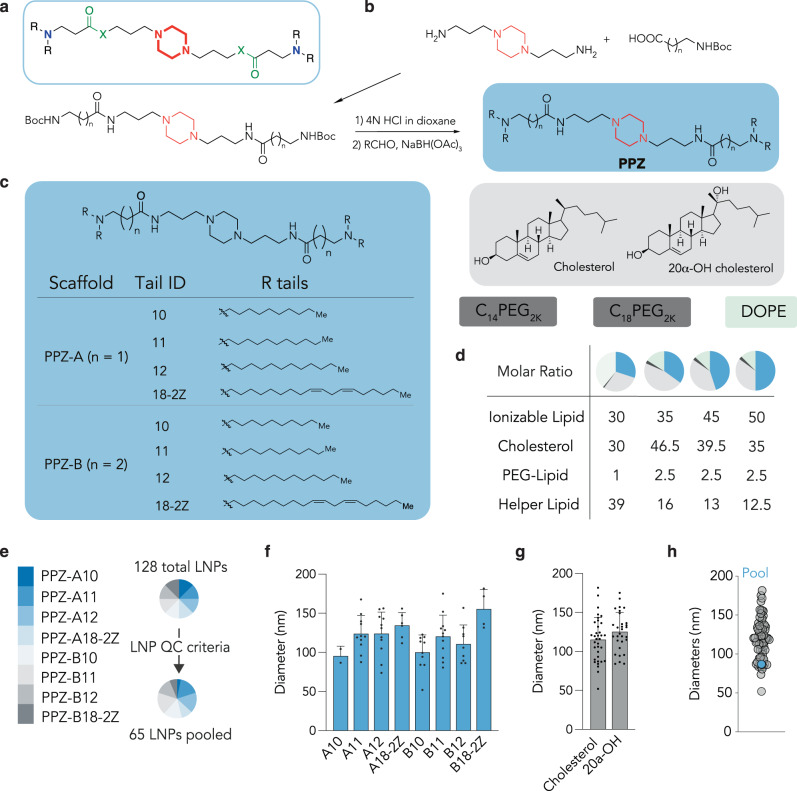
Piperazine-based lipids formulate into stable lipid nanoparticles (LNPs). **a** Design of ionizable lipids with piperazine backbone. **b** Procedure to synthesize PPZ lipids. **c** Composition of the LNP library: 8 different PPZ lipids, 2 cholesterol variants (cholesterol and 20α-OH cholesterol), 2 PEG-lipid variants (C_14_PEG_2K_ and C_18_PEG_2K_), and DOPE. **d** Each of the compounds was formulated using four molar ratios. **e** Of the 128 LNPs that were formulated, 65 passed the quality control criteria, with a diameter less than 200 nm as well as a stable autocorrelation curve. **f** Hydrodynamic diameters of LNPs formulated with PPZ lipids, average ± SEM. **g** Hydrodynamic diameters of LNPs formulated with cholesterol and 20α-OH cholesterol, average ± SEM. **h** Hydrodynamic diameters of all administered LNPs; the diameter of the LNP pooled control is within the range of the LNPs composing the pool.

We first rationally designed ionizable lipids consisting of a piperazine core and two tertiary amines as ionizable headgroups linked to hydrophobic carbon chains, which we termed “Pi-Lipids” (Fig. 1a). We originally chose ester bonds as linkers; however, this synthetic strategy did not afford the expected compounds. We therefore switched to an amide bond. To the piperazine core, we added a saturated hydrocarbon chain ranging from C10 to C12 in our design. We chose this lipid length since C8 to C12 lipids are well represented among other lead ionizable lipids^26, 39^ and may help to disrupt cellular membranes, thereby facilitating delivery^40^. Finally, since it was shown that linoleate chains enhanced LNP delivery^41^, we added linoleate-based scaffolds to our design. We successfully synthesized eight novel piperazine-based ionizable lipids (Fig. 1b, c). Briefly, a simple and straightforward amide coupling reaction between 1,4-bis(3-aminopropyl)piperazine and Boc-protected β-Alanine or γ-Aminobutyric acid yielded piperazine intermediates, in 12 h, with a 50% yield. A subsequent Boc deprotection followed by a one-pot reductive amination reaction with different hydrophobic aldehydes led to the final piperazine-based lipids (PPZ) in yields of 32 to 59%. We varied the length of the carbon chain linkage and synthesized lipids in two scaffolds, PPZ-A containing two carbons as linkage and PPZ-B containing three carbons. The lipid structures were confirmed by nuclear magnetic resonance (NMR) and high-resolution mass spectrometry (HRMS) (see supporting information) (Fig. 1b).

### PPZ lipids formulate into stable lipid nanoparticles with mRNA

We then investigated whether novel Pi-Lipids formulated into stable, monodisperse LNPs, which we termed Pi-LNPs. LNPs are typically formulated using four components: (i) an ionizable or cationic lipid, (ii) a PEG-lipid, (iii) a cholesterol, and (iv) a helper lipid. Thus, to isolate the effect of the Pi-Lipids, we formulated the LNPs with components that were previously shown to form stable LNPs with other (i) ionizable or cationic lipids. Specifically, we chose (ii) two PEG-lipids with different lengths of carbon chains (C_14_PEG_2K_ and C_18_PEG_2K_), (iii) two cholesterol variants (cholesterol, 20α-OH cholesterol), and (iv) 1,2-dioleoyl-sn-glycerol-3-phosphoethanolamine (DOPE) (Fig. 1b, c). Since LNP formation and stability can vary with the ratio of these four components, we added a control to ensure our results were robust, testing four different molar ratios (Fig. 1d). Thus, using microfluidics^42^, we formulated 128 chemically distinct Pi-LNPs.

We analyzed Pi-LNPs using a FIND^32^, a DNA barcode-based assay that quantifies how dozens of different LNPs deliver mRNA in vivo^43–45^. We formulated LNP 1, with chemical composition 1, to carry Cre mRNA and DNA barcode 1, and LNP N, with chemical composition N, to carry Cre mRNA and DNA barcode N. By incorporating a distinct DNA barcode in each LNP, we were able to identify individual LNP delivery through deep sequencing. We performed a quality control step, quantifying the hydrodynamic diameter and polydispersity of all 128 Pi-LNPs individually using dynamic light scattering. Only monodisperse Pi-LNPs with diameters from 20 nm to 200 nm, measured by intensity average, were selected. Of the original 128 Pi-LNPs, 65 met these criteria and were pooled together for subsequent in vivo administration (Fig. 1d, e).

To understand the effect of chemical structure on Pi-LNP formation, we analyzed the hydrodynamic diameters, observing that lipids with shorter C10 carbon chains formed Pi-LNPs with an average diameter of 96 nm for PPZ-A10 and 100 nm for PPZ-B10. By contrast, lipids with longer carbon chains (C_18_) formed LNPs with larger diameters, including 134 nm for PPZ-A18-2Z and 155 nm for PPZ-B18-2Z (Fig. 1f). We found that while the Pi-LNP diameter did seem to vary with the structure of the Pi-Lipid, the diameter did not change as a function of the cholesterol (Fig. 1g). Finally, we tested the diameter of the pool of Pi-LNPs and found it within the range of the diameters of the 65 Pi-LNPs, suggesting that mixing the Pi-LNPs did not cause them to come out of the solution (Fig. 1h). Taken together, these data led us to conclude that Pi-Lipids can form monodisperse, stable Pi-LNPs.

**Fig. 2:**
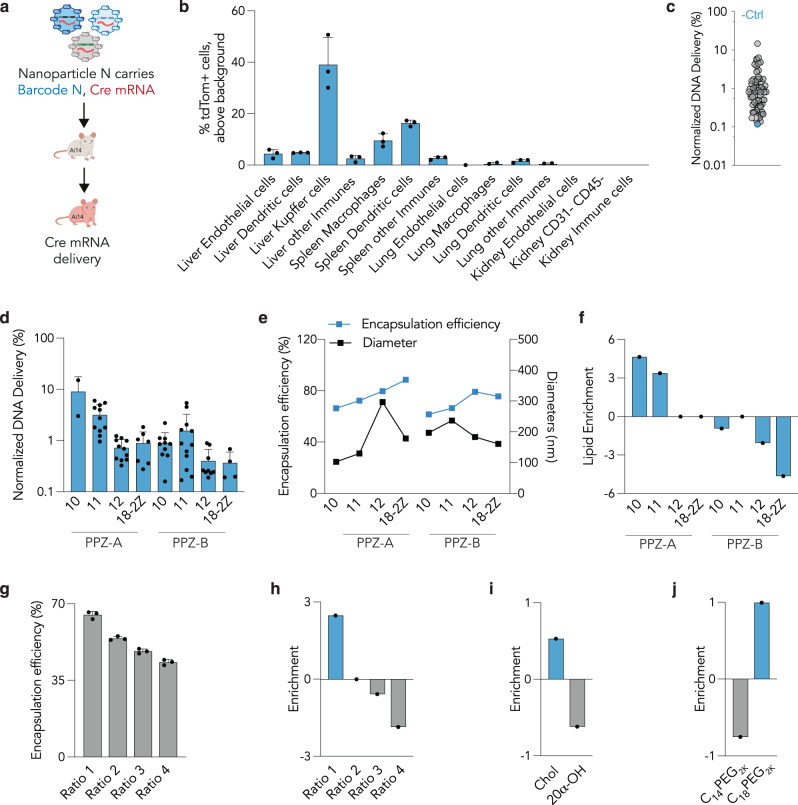
Quantifying how 65 LNPs delivered mRNA delivery to 14 cell types in vivo, and subsequent in vivo structure-function analysis. **a** LNPs were formulated to carry a unique DNA barcode and Cre mRNA. The 65 LNP pool was then administered to Ai14 mice. After 3 days %tdTomato+ cells were quantified in **b** multiple cell types in the liver, spleen, lung, and kidney (Average ± SEM, *N* = 4/group). Source data are provided as a Source Data file. **c** Normalized delivery for all 65 LNPs, averaged across all samples. Unencapsulated DNA barcode, acting as a negative control (-Ctrl), was delivered into cells less efficiently than barcodes encapsulated by LNPs. **d** Normalized delivery of LNPs formulated with each PPZ lipid, average ± SD. **e** Encapsulation efficiencies and diameters for LNPs formulated with PPZ-A10, cholesterol, C18PEG2K, and DOPE at a ratio of 35:46.5:2.5:16. **f** Fold enrichment calculated based on different lipids. **g** Encapsulation efficiencies of LNPs formulated with PPZ-A10, cholesterol, C_18_PEG_2K_, DOPE at four molar ratios; ratio 1 = 30:30:1:39; ratio 2 = 35:46.5:2.5:16; ratio 3 = 45:39.5:2.5:13; ratio 4 = 50:35:2.5:12.5, average ± SEM. **h** Fold enrichment calculated based on different ratios. **i** Fold enrichment calculated for cholesterol and 20□-OH cholesterol. **j** Fold enrichment calculated for C_14_PEG_2K_ and C_18_PEG_2K_.

After characterizing the pool of 65 Pi-LNPs, we intravenously injected them in Ai14 mice at a total nucleic dose of 1.5 mg/kg (averaging a 0.023 mg total nucleic acid/kg/particle, for all 65 Pi-LNPs) (Fig. 2a). The Ai14 mice have a Lox-Stop-Lox-tdTomato construct downstream of a CAG promoter. Thus, if Cre mRNA is delivered into a target cell and is subsequently functionally translated into Cre protein, the cells become tdTomato + (Fig. 2a). By isolating tdTomato+ cells using fluorescence-activated cell sorting (FACS) (Supplementary Figs. 1 and 2) and sequencing the cells using next-generation sequencing, we can isolate the DNA barcodes, associated with specific LNPs, within cells that were functionally transfected with Cre mRNA^32, 43–45^. Three days after injection, we isolated the liver, spleen, lung, and kidney and quantified the percentage of tdTomato+ cells from 14 different cell populations (Fig. 2b). We observed 40% of tdTomato+ cells in Kupffer cells, 10% in spleen macrophages, and 16% in spleen dendritic cells. The percentage of tdTomato+ quantified in liver endothelial cells and dendritic cells was < 5%, and we did not observe any delivery in the lung and kidney. After isolating tdTomato+ cells from the most targeted cell populations—Kupffer cells, spleen macrophages, and dendritic cells—we investigated how well each of the 65 LNPs performed using next-generation DNA sequencing. From the barcode raw counts obtained through sequencing, we calculated the normalized delivery of each individual barcode. Briefly, the normalized delivery of a given barcode is calculated as the number of counts for that barcode divided by the counts for all N barcodes (Supplementary Fig. 3). This allowed us to identify barcodes that were preferentially delivered to specific cell types, which then directed us to LNPs carrying those barcodes. As a control, we quantified unencapsulated barcodes, which were also injected. Since unprotected DNA does not readily enter cells, its normalized delivery is expected to be the lowest among all the barcodes^30^, which was the case when the barcodes were calculated across all cell types (Fig. 2c).

### Understanding LNP properties and tropism with PPZ lipids

We then used this large dataset to perform a comprehensive in vivo structure-function analysis across all cell types. First, we analyzed the averaged normalized delivery of LNPs based on different Pi-Lipid structures and found that Pi-LNPs containing PPZ-A10 exhibited the highest delivery, followed by Pi-LNPs formulated with PPZ-A11 (Fig. 2d). We hypothesized that the difference in normalized delivery observed between Pi-LNPs could be due to disparities in encapsulation efficiency or LNP diameters. To test this hypothesis, we formulated eight LNPs varying only the ionizable lipid structure while keeping the same molar ratio and compound compositions and measured both the diameter and encapsulation efficiency for each LNP (Fig. 2e). Interestingly, we observed an increase in the encapsulation efficiency from 66 to 88% for Pi-LNPs formulated with PPZ-A10 to PPZ-A18-2Z, respectively, suggesting that the encapsulation efficiency increases with longer carbon chains. However, Pi-LNPs with longer carbon chains also displayed large diameters between 150 and 300 nm, which is unfavorable for LNP delivery^46^. Encapsulation efficiencies between Pi-LNPs formulated with PPZ-A and PPZ-B lipids were comparable, but large diameters were observed for Pi-LNPs containing PPZ-B lipids, potentially explaining the reduced delivery observed for those compounds (Fig. 2d). To complement this structure-function analysis, which included all tested Pi-LNPs, we analyzed the relationship between Pi-Lipid structure and in vivo activity using enrichment (Fig. 2f), which only includes the best and worst nanoparticles. Enrichment, which can be used to understand LNP structure function^47, 48^, calculates the odds that a material is found in the top or bottom 10% of the LNPs, relative to random chance (Supplementary Fig. 4). Consistent with the normalized delivery data, enrichment analysis highlighted that PPZ-A scaffolds outperformed PPZ-B, and among all lipids, PPZ-A10 was the most enriched. Subsequently, we investigated how other material properties affected LNP delivery. We formulated four LNPs using PPZ-A10, cholesterol, C_18_PEG_2K_, and DOPE, varying only the molar ratio of each component, and measured the encapsulation efficiencies (Fig. 2g). Ratio 1, containing the lowest percentage of ionizable lipid and the highest percentage of DOPE, formulated with the highest encapsulation efficiency, whereas increasing the molar ratio of ionizable lipid, and therefore decreasing DOPE, resulted in a reduction of encapsulation efficiency. We then explored how these molar ratios affected normalized delivery by calculating the fold of enrichment for each ratio (Fig. 2h). Once again, ratio 1 was the most enriched and ratio 4 was the most depleted. We therefore concluded that a lower molar ratio of ionizable lipid and a higher percentage of DOPE was optimal for PPZ-based LNP formulation in this study. We also analyzed the fold of enrichment for different cholesterol variants and PEG lipids and found that cholesterol outperformed 20α-OH cholesterol (Fig. 2i), and C_18_PEG_2K_ outperformed C_14_PEG_2K_ (Fig. 2j). Based on these data, we reached two conclusions: first, that PPZ-A scaffolds outperformed PPZ-B scaffolds; and second, that PPZ-A10, cholesterol, and C_18_PEG_2K_ could promote delivery to the liver and spleen, relative to the other components we investigated.

**Fig. 3:**
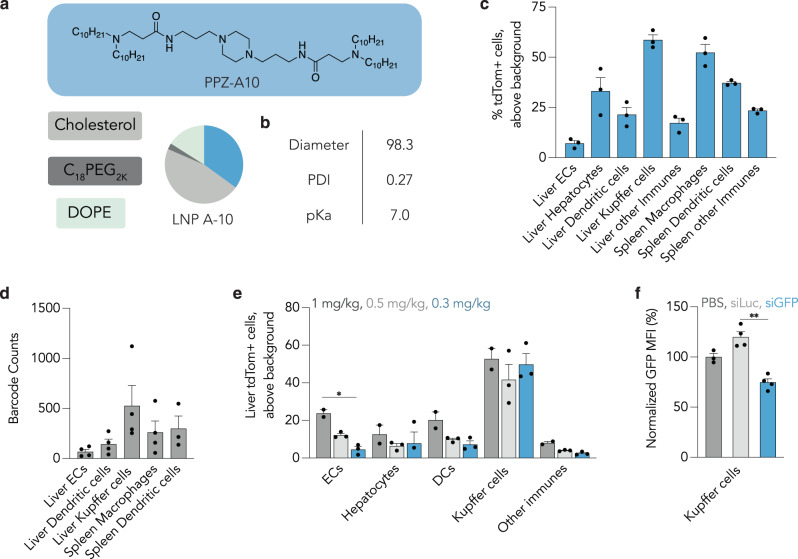
LNPs containing piperazine-based lipids deliver mRNA to immune cells. **a** A top-performing LNP-A10 with PPZ-A10, cholesterol, C_18_PEG_2K_ and DOPE at a ratio of 35:46.5:2.5:16 was identified and formulated with Cre mRNA. **b** The diameter (nm), polydispersity index (PDI), and pKa of LNP-A10. **c** LNP-A10 was injected to Ai14 mice at a dose of 1 mg/kg, and %tdTomato+ cells in liver endothelial cells (ECs), hepatocytes, dendritic cells, Kupffer cells, other immunes and spleen macrophages, spleen dendritic cells and spleen other immunes were quantified after three days. *N* = 3/group, average + SEM. Source data are provided as a Source Data file. **d** Biodistribution of LNP-A10 in liver ECs, dendritic cells, Kupffer cells, spleen macrophages and spleen dendritic cells. *N* = 4/group, average ± SEM. Source data are provided as a Source Data file. **e** %tdTomato+ cells in liver ECs, hepatocytes, dendritic cells, Kupffer cells and liver other immunes after treatment of LNP-A10 at doses of 1 mg/kg, 0.5 mg/kg, and 0.3 mg/kg. *N* = 3/group, average ± SEM. Two-way ANOVA, *P = 0.0083; ns: not significant. Source data are provided as a Source Data file. **f** Normalized GFP MFI in Kupffer cells after treatment of LNP-A10 carrying siGFP and siLuc at a dose of 1 mg/kg. *N* = 4/group, average ± SEM. Two-way ANOVA, **P = 0.0075. Source data are provided as a Source Data file.

Based on our in vivo structure-function analysis, we identified a top Pi-LNP, named LNP-A10 (Fig. 3a, b), which contains the ionizable lipid PPZ-A10, cholesterol, C_18_PEG_2K_, and DOPE at a ratio of 35:46.5:2.5:16. To validate LNP-A10, we formulated it with Cre mRNA and injected it intravenously into Ai14 mice at a dose of 1 mg/kg. Mouse weights were monitored throughout the experiment, and no weight loss was observed (Supplementary Fig. 5). After three days, we isolated cells of interest and evaluated the percentage of tdTomato+ cells at the cell-type level (Fig. 3c). LNP-A10 successfully delivered Cre mRNA predominantly to (1) Kupffer cells, with 60% tdTomato+ cells observed, (2) spleen macrophages, with 50% tdTomato+ cells, and (3) spleen dendritic cells, with 30% tdTomato+ cells. We also observed 20% delivery to liver dendritic cells, while the delivery to liver endothelial cells was below 10%. To complement the tdTomato readouts, which quantify the functional delivery of mRNA, we measured the biodistribution of LNP-A10 using QUANT^49^, a sensitive digital droplet PCR-based method to quantify on- and off-target biodistribution (Fig. 3d). Once again, the distribution of LNP-A10 was found to be the highest in Kupffer cells, followed by spleen dendritic cells and macrophages, which was consistent with the functional delivery results we observed. We therefore concluded that LNP-A10 preferentially delivered nucleic acids to hepatic and splenic immune cells.

Next, we performed an in vivo dose response to explore whether LNP-A10 delivered mRNA at 0.3 mg/kg, a clinically relevant dose^1^. We injected LNP-A10 including Cre mRNA at doses of 1 mg/kg, 0.5 mg/kg and 0.3 mg/kg (Fig. 3e). At the lowest dose, we observed 50% tdTomato^+^ Kupffer cells, 23% tdTomato^+^ splenic macrophages, and 26% tdTomato^+^ splenic dendritic cells, demonstrating that LNP-A10 can deliver mRNA-relevant payloads. Finally, we evaluated whether LNP-A10 delivered siRNA; notably, it can be difficult to identify a single nanoparticle that efficiently delivers both mRNA and siRNA, due to the distinct biophysical differences between the two payloads^50^. We therefore formulated LNP-A10 with siGFP as well as siLuciferase (siLuc) and injected it intravenously into GFP mice at a dose of 1 mg/kg. siLuc, an siRNA that does not interfere with GFP expression, was included as a control to eliminate the possibility of a toxicity-induced decrease in GFP protein expression. We observed about 25% silencing of GFP protein expression in Kupffer cells (Fig. 3f), whereas no silencing was observed in control mice injected with siLuc. This led us to conclude that LNP-A10 could also deliver siRNA, albeit with lower efficiency than mRNA.

## Discussion

By designing, synthesizing, and characterizing 128 novel Pi-LNPs, we found that Pi-Lipids can be formulated into stable nanoparticles, and that these nanoparticles can deliver nucleic acids to non-hepatocytes in vivo. Notably, the leading LNP, LNP-A10, that delivered mRNA preferentially to liver and spleen immune cells at a dose as low as 0.3 mg/kg, was identified directly using an in vivo barcoding approach, demonstrating the utility of direct to in vivo high-throughput nanoparticle studies. We compared the structure of our PPZ-lipids with lipids that included a piperazine motif. When compared to C12-200^39^, we added amide bonds and removed hydroxyl groups. These hydroxyl can lead to stereoisomers, which can make purification difficult. By contrast, the PPZ-lipids are stereopure, which makes them easier to purify. These chemical changes may be responsible for the shift in tropism we noted; specifically, we found increased splenic macrophage and dendritic cell delivery, compared to previously reported C12-200 publications. We then compared the structure to C14-4, which was shown to deliver mRNA to Jurkat cells and primary human T cells in vitro^51^. However, it is difficult to compare in vitro and in vivo delivery^29^. One key limitation of this work is that LNP delivery may vary across species^52^, and thus, these results need to be confirmed in larger animals. Taken together, we believe the data justify further exploration of LNPs with piperazine rings.

## Methods

### Statistics & reproducibility

For in vivo experiments: *N* = 2 for PBS negative control and 3 or 4 mice for experimental control. Those sample sizes were chosen to ensure the accuracy of the data and accurate statistics. The 2 mice per PBS control were chosen to limit the number of mice. No statistical method was used to predetermine sample size. No data were excluded from the analyses. Mice were randomly selected, and no algorithm was used. The Investigators were not blinded to allocation during experiments and outcome assessment.

### Nanoparticle formulation

Nanoparticles were formulated with a microfluidic device as previously described^1^. Nucleic acids (DNA barcodes and mRNA) were diluted in a 10 mM citrate buffer (Teknova). Lipid-amine compounds, PEG-lipids (1,2-dimyristoyl-sn-glycerol-3-phosphoethanolamine-N-[methoxy(polyethyleneglycol)−2000] and 1,2-distearoyl-sn-glycerol-3-phosphoethanolamine-N-[methoxy(polyethyleneglycol)−2000]), cholesterols (cholesterol and 20α-OH cholesterol), and helper lipid 1,2-dioleoyl-sn-glycerol-3-phosphoethanolamine were diluted in 100% ethanol. Cre mRNA and DNA barcodes were mixed at a 10:1 mass ratio for mRNA screens. All PEGs, cholesterols, and helper lipids were purchased from Avanti Lipids. Citrate and ethanol phases were combined in a microfluidic device by syringes (Hamilton Company) at a flow rate of 3:1. The LNPs were dialyzed into 1× PBS to remove the solvent.

### DNA barcoding

Each LNP was formulated to carry its own unique DNA barcode. DNA barcodes were designed rationally with several characteristics as described^2^. All DNA barcodes were 91-nt-long, single-stranded DNA sequences purchased from Integrated DNA Technologies. Briefly, the following modification was on all barcodes: (i) nucleotides on the 5′ and 3′ ends were modified with phosphorothioates to reduce exonuclease degradation (ii) universal forward and reverse primer regions were included to ensure equal amplification of each sequence, (iii) 7 random nucleotides were included to monitor PCR bias, (iv) a droplet digital PCR (ddPR) probe site was included for ddPCR compatibility, and (v) a unique 8-nt barcode was assigned. An 8-nucleotide sequence can generate over 4 (65,536) distinct barcodes. We used only the 8-nucleotide sequences designed to prevent sequence bleaching and reading errors on the Illumina MiniSeq^TM^ sequencing machine.

### Nanoparticle characterization

LNP hydrodynamic diameter was measured using high-throughput dynamic light scattering (DynaPro Plate Reader II, Wyatt). LNPs were diluted in sterile 1× PBS and analyzed. To avoid using unstable LNPs, and to enable sterile purification using a 0.22 μm filter, LNPs were included only if they met three criteria: diameter >20 nm, diameter <200 nm, and correlation function with 1 inflection point. Particles that met these criteria were pooled and dialyzed in a 20 kD dialysis cassette (Thermo Scientific) and a 100 kD cassette (Thermo Scientific) in 1X PBS. The nanoparticle concentration was determined using NanoDrop (Thermo Scientific). LNP encapsulation was measured using a Quant-iT RiboGreen assay (Thermo Fisher)^3^.

### Animal experiments

All animal experiments were performed in accordance with the Georgia Institute of Technology’s IACUC (protocol number A100238). All animals were bred in the Georgia Institute of Technology Animal Facility. C57BL/6 J (#000664) were purchased from The Jackson Laboratory. LSL-Tomato/Ai14 (#007914) were purchased from The Jackson Laboratory for breeding purposes. All mice were 6 to 8 weeks old at the time of the experiments. In all experiments, we used *N* = 2–4 mice/group. Mice were injected intravenously via the lateral tail vein. The nanoparticle concentration was determined using NanoDrop (Thermo Scientific). All animals were housed in the Georgia Institute of Technology Animal Facility.

### Cell isolation and staining

Cells were isolated 24 or 72 h after injection with LNPs, unless otherwise noted. Mice were perfused in the liver portal vein with 5 mL of Krebs Ringer buffer (pH 7.3). Tissues were finely minced and then placed in Collagenase XI (Sigma Aldrich) at 37 °C at 500 rpm for 30 min. The cell suspension was filtered through 70 μm mesh and washed with 1× PBS. Cells were stained to identify specific cell populations and sorted using the BD FacsFusion cell sorter at the Georgia Institute of Technology Cellular Analysis Core. The antibody clones used were: Live/dead far-red fluorescent dye (Invitrogen, dilution 1:250), anti-mouse CD31 (390, BioLegend, dilution 1:250), anti-mouse CD45.2 (104, BioLegend, dilution 1:250), anti-mouse CD68 (FA11, BioLegend, dilution 1:250), anti-mouse CD11b (M1/70, BioLegend, dilution 1:250), anti-mouse CD11c (N418, BioLegend, dilution 1:250), anti-mouse CD3 (17A2, Biolegend, dilution 1:250), anti-mouse CD19 (6D5, BioLegend, dilution 1:250). Representative flow gates are in Supplementary Figs. 1 and 2. PBS-injected mice were used to gate on tdTomato positive populations.

### PCR amplification

All samples were amplified and prepared for sequencing using nested PCR^4^. More specifically, 1 μL of primers (5 uM for Final Reverse/Forward) were added to 5 μL of Kapa HiFi 2× master mix (Roche), and 4 μL template DNA/water. During the second PCR Nextera XT chemistry, indices and i5/i7 adapter regions were added. Dual-indexed samples were run on a 2% agarose gel to ensure that PCR reaction occurred before being pooled and gel purified. The primers used for the nested PCR are listed below, all primers were mixed in equal molar:

**Table:** 

FWD_1	TCG TCG GCA GCG TCA GAT GTG TAT AAG AGA CAG GCT CTC ATA CGA ACT CGT CC
FWD_2	TCG TCG GCA GCG TCA GAT GTG TAT AAG AGA CAG TGC TCT CAT ACG AAC TCG TCC
FWD_3	TCG TCG GCA GCG TCA GAT GTG TAT AAG AGA CAG ATG CTC TCA TAC GAA CTC GTC C
FWD_4	TCG TCG GCA GCG TCA GAT GTG TAT AAG AGA CAG GAT GCT CTC ATA CGA ACT CGT CC
FWD_5	TCG TCG GCA GCG TCA GAT GTG TAT AAG AGA CAG CGA TGC TCT CAT ACG AAC TCG TCC
FWD_6	TCG TCG GCA GCG TCA GAT GTG TAT AAG AGA CAG TCG ATG CTC TCA TAC GAA CTC GTC C
FWD_7	TCG TCG GCA GCG TCA GAT GTG TAT AAG AGA CAG ATC GAT GCT CTC ATA CGA ACT CGT CC
FWD_8	TCG TCG GCA GCG TCA GAT GTG TAT AAG AGA CAG GAT CGA TGC TCT CAT ACG AAC TCG TCC
RVS_1	GTC TCG TGG GCT CGG AGA TGT GTA TAA GAG ACA GGT CTC TGC TCG ACT AAC CAC
RVS_2	GTC TCG TGG GCT CGG AGA TGT GTA TAA GAG ACA GTG TCT CTG CTC GAC TAA CCA C
RVS_3	GTC TCG TGG GCT CGG AGA TGT GTA TAA GAG ACA GAT GTC TCT GCT CGA CTA ACC AC
RVS_4	GTC TCG TGG GCT CGG AGA TGT GTA TAA GAG ACA GGA TGT CTC TGC TCG ACT AAC CAC
RVS_5	GTC TCG TGG GCT CGG AGA TGT GTA TAA GAG ACA GCG ATG TCT CTG CTC GAC TAA CCA C
RVS_6	GTC TCG TGG GCT CGG AGA TGT GTA TAA GAG ACA GTC GAT GTC TCT GCT CGA CTA ACC AC
RVS_7	GTC TCG TGG GCT CGG AGA TGT GTA TAA GAG ACA GAT CGA TGT CTC TGC TCG ACT AAC CAC
RVS_8	GTC TCG TGG GCT CGG AGA TGT GTA TAA GAG ACA GGA TCG ATG TCT CTG CTC GAC TAA CCA C

### Deep sequencing

PCR samples were purified by AMPure XP beads. Final library QC was conducted using the Agilent Bioanalyzer 2100. Illumina deep sequencing was conducted on an Illumina MiniSeq^TM^. Primers were designed based on Nextera XT adapter sequences.
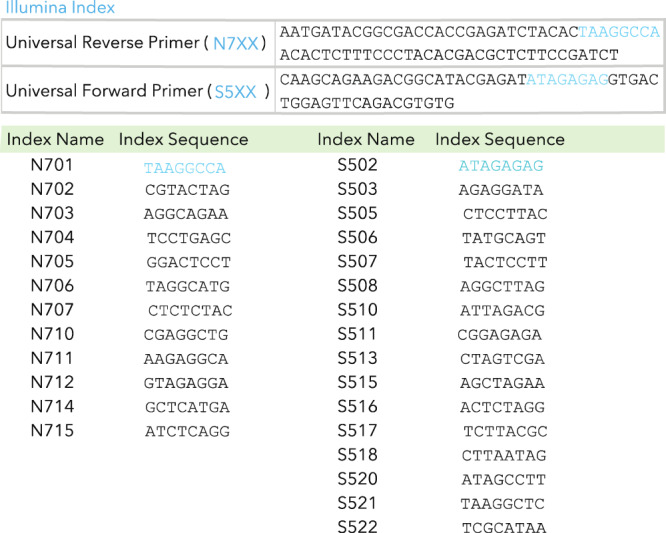


### Nanoparticle data analysis and statistics

Sequencing results were processed using a custom Python-based tool to extract raw barcode counts for each tissue. These raw counts were normalized with an R script before further analysis. Counts for each particle, per tissue, were normalized to the barcoded LNP mixture injected into the mouse. This “input” DNA provided the DNA counts and was used to normalize DNA counts from the cells and tissues. Statistical analysis was done using GraphPad Prism 8. Data are plotted as mean ± standard error mean unless otherwise stated.

### Reporting summary

Further information on research design is available in the Nature Research Reporting Summary linked to this article.

## Supplementary information

Supplementary Information

Reporting Summary

## Data Availability

Source data are provided with this manuscript. The scripts used to analyze DNA barcoding results are available online [https://github.com/Jack-Feldman/barcode_count]. All other data are shown in the manuscript and Supporting Information. H.N., M.Z.C.H., and J.E.D. via the Georgia Institute of Technology Research Corporation, have filed a provisional patent application related to this technology (US patent provisional 63/293,287). Source data are provided with this paper.

## Source data

Source Data

## Code availability

All code used to analyze the data is available online [https://github.com/Jack-Feldman/barcode_count].
