# Supplementary material for: Piperazine-derived lipid nanoparticles deliver mRNA to immune cells in vivo
Source: Nat Commun. 2022 Aug 15;13:4766. doi: 10.1038/s41467-022-32281-5 (PMC9376583; doi:10.1038/s41467-022-32281-5)
Supplement: Supplementary file 1 — Supplementary Information [file 41467_2022_32281_MOESM1_ESM.pdf]

Piperazine-derived lipid nanoparticles deliver mRNA to immune cells *in vivo*

Huanzhen Ni[1,+], Marine Z.C. Hatit[1,+], Kun Zhao[1,2,+], David Loughrey[1], Melissa P. Lokugamage [1], Hannah E. Peck[3], Ada Del Cid[1], Abinaya Muralidharan[4,5], YongTae Kim[1,3,4,5], Philip J. Santangelo[1], James E. Dahlman[1,*]

[1]Wallace H. Coulter Department of Biomedical Engineering, Georgia Institute of Technology, Atlanta, GA, 30332, USA

[2]Current address: School of Pharmaceutical Sciences, Shandong University, Jinan, China

[3]Parker H. Petit Institute for Bioengineering and Bioscience, Georgia Institute of Technology, Atlanta, GA, 30332, USA

[4]George W. Woodruff School of Mechanical Engineering, Georgia Institute of Technology, Atlanta, GA, 30332, USA

[5]Institute for Electronics and Nanotechnology, Georgia Institute of Technology, Atlanta, GA, 30332, USA

[+]H.N., M.Z.C.H., and K.Z. contributed equally to this work.

*Correspondence: james.dahlman@bme.gatech.edu

A. Synthesis and Characterization of Intermediates and Ionizable Lipids S2

B. Supplementary Figures S5

C. Reference S8

D. NMR and HRMS Spectra of Intermediates and Ionizable Lipids S9

A. Synthesis and Characterization of Ionizable Lipids

A.1 Synthesis of intermediate

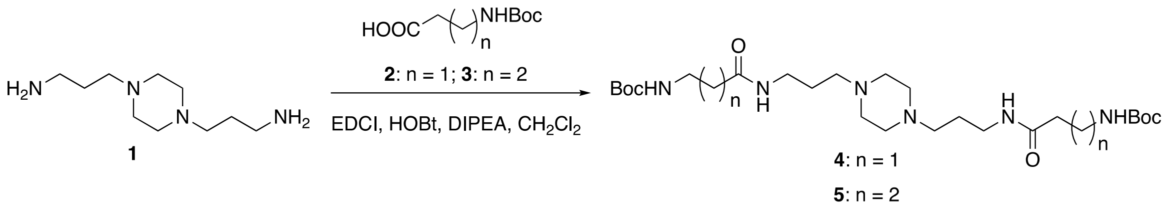

To a mixture of **2** or **3** (4.5 mmol) in 10 mL CH_2Cl_2 was added DIPEA (4.5 mmol), followed by EDCI (4.5 mmol) and HOBt (4.5 mmol). The mixture was stirred at room temperature for 10 min and then was added **1** (1.5 mmol) dropwise. The resulting mixture was stirred at room temperature for 12 hr and quenched by the addition of saturated $NaHCO_3$ solution (20 mL). The aqueous phase was extracted with CH_2Cl_2 (20 mL) three times and concentrated in vacuo. The crude product was then purified by column chromatography using eluent CH_2Cl_2/MeOH (10:1).

4: white solid, yield 50%. ^{1}H NMR (500 MHz, $CDCl_3$) δ 7.18 (s, 2H), 5.24 (t, *J* = 6.0 Hz, 2H), 3.39 (q, *J* = 6.2 Hz, 4H), 3.33 (q, *J* = 6.1 Hz, 4H), 2.57 – 2.42 (m, 8H), 2.35 (t, *J* = 6.0 Hz, 4H), 2.01 – 1.83 (m, 4H), 1.67 (p, *J* = 6.3 Hz, 4H), 1.42 (s, 18H). ^{13}C NMR (125 MHz, $CDCl_3$) δ 171.18, 156.12, 79.24, 57.25, 53.22, 50.82, 39.37, 36.73, 36.36, 28.42, 24.94. HRMS (ESI) m/z calcd for C26H50N6O6 $[M+H]^+$= 543.3864, found = 543.3867.

5: white solid, yield 47%. ^{1}H NMR (500 MHz, $CDCl_3$) δ 7.21 (t, *J* = 5.3 Hz, 2H), 4.90 (t, *J* = 6.5 Hz, 2H), 3.30 (q, *J* = 5.7 Hz, 4H), 3.13 (q, *J* = 6.6 Hz, 4H), 2.54 – 2.40 (m, 8H), 2.17 (t, *J* = 7.2 Hz, 4H), 1.78 (p, *J* = 7.0 Hz, 4H), 1.67 (p, *J* = 6.6 Hz, 4H), 1.41 (s, 18H). ^{13}C NMR (125 MHz, $CDCl_3$) δ 172.51, 156.41, 79.23, 57.06, 53.27, 50.65, 39.94, 39.10, 33.95, 28.43, 26.31, 25.31. HRMS (ESI) m/z calcd for C28H54N6O6 $[M+H]^+$= 571.4177, found = 571.4179.

A.2 Synthesis of PPZ ionizable lipids

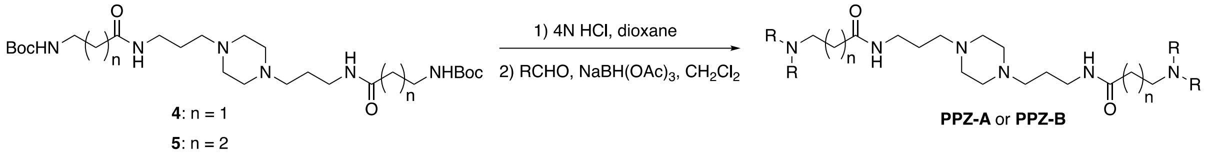

To the intermediate **4** or **5** (0.1 mmol) was added 3 mL 4N HCl in dioxane at 0 °C and the mixture was stirred at room temperature for 1 hr. Solvent was removed in vacuo and the crude product was suspended in 1 mL CH_2Cl_2. Aldehyde RCHO (0.6 mmol) was added followed by $NaBH(OAc)_3$ (0.5 mmol). The solid suspension started to dissolve with the progress of reaction. The resulting mixture was stirred at room temperature for 12 hr and quenched by addition of 1 mL saturated $NaHCO_3$ solution. The product was then purified by column chromatography using CH_2Cl_2/MeOH (10:1).

PPZ-A10: colorless oil, yield 56%. ^{1}H NMR (500 MHz, $CDCl_3$) δ 8.52 (t, *J* = 5.6 Hz, 2H), 3.23 (q, *J* = 6.6 Hz, 4H), 2.63 (t, *J* = 6.1 Hz, 4H), 2.57 – 2.24 (m, 24H), 1.70 – 1.61 (m, 4H), 1.46 – 1.36 (m, 8H), 1.30 – 1.18 (m, 54H), 0.86 (t, J = 6.9 Hz, 12H). ^{13}C NMR (125 MHz, $CDCl_3$) δ 172.66, 56.33, 53.25, 53.22, 50.26, 37.52, 32.74, 31.84, 29.62, 29.54, 29.27, 27.58, 26.72, 26.49, 22.62, 14.06. HRMS (ESI) m/z calcd for C56H114N6O2 $[M+2H]^{2+}$= 452.4574, found = 452.4570.

PPZ-A11: colorless oil, yield 59%. ^{1}H NMR (500 MHz, $CDCl_3$) δ 8.52 (t, *J* = 5.6 Hz, 2H), 3.23 (q, *J* = 6.7 Hz, 4H), 2.63 (t, *J* = 6.1 Hz, 4H), 2.56 – 2.21 (m, 24H), 1.65 (q, *J* = 7.2 Hz, 4H), 1.47 – 1.36 (m,

10H), 1.31 – 1.17 (m, 66H). ^{13}C NMR (125 MHz, $CDCl_3$) δ 172.69, 56.40, 53.31, 53.29, 50.32, 37.59, 32.82, 31.91, 29.68, 29.65, 29.63, 29.60, 29.35, 28.40, 27.64, 26.79, 26.56, 22.69, 14.13. HRMS (ESI) m/z calcd for C60H122N6O2 $[M+2H]^{2+}$= 480.4887, found = 480.4886.

PPZ-A12: colorless oil, yield 47%. ^1H NMR (500 MHz, $CDCl_3$) δ 8.50 (t, *J* = 5.6 Hz, 2H), 3.23 (q, *J* = 6.7 Hz, 4H), 2.64 (t, *J* = 6.1 Hz, 4H), 2.59 – 2.20 (m, 24H), 1.65 (p, *J* = 7.1 Hz, 4H), 1.48 – 1.33 (m, 9H), 1.32 – 1.11 (m, 75H), 0.85 (t, *J* = 6.9 Hz, 12H). ^{13}C NMR (125 MHz, $CDCl_3$) δ 172.66, 56.40, 53.30, 53.28, 50.31, 37.60, 32.80, 31.92, 29.68, 29.65, 29.60, 29.36, 27.63, 26.76, 26.52, 22.69, 14.12. HRMS (ESI) m/z calcd for C64H130N6O2 $[M+2H]^{2+}$= 508.5200, found = 508.5201.

PPZ-A18-2Z: colorless oil, yield 38%. ^1H NMR (500 MHz, $CDCl_3$) δ 8.45 (t, *J* = 5.7 Hz, 2H), 5.43 – 5.19 (m, 16H), 3.23 (q, *J* = 6.7 Hz, 4H), 2.75 (t, *J* = 6.7 Hz, 8H), 2.66 (t, *J* = 6.1 Hz, 4H), 2.50 – 2.28 (m, 24H), 2.03 (q, *J* = 7.0 Hz, 16H), 1.71 – 1.61 (m, 4H), 1.46 – 1.38 (m, 10H), 1.37 – 1.21 (m, 62H), 0.87 (t, *J* = 6.9 Hz, 12H). ^{13}C NMR (125 MHz, $CDCl_3$) δ 172.61, 130.21, 130.04, 128.04, 127.90, 56.40, 53.30, 53.27, 50.25, 37.64, 32.81, 31.53, 29.67, 29.60, 29.58, 29.35, 29.29, 27.64, 27.23, 27.21, 26.75, 26.46, 25.64, 22.58, 14.10. HRMS (ESI) m/z calcd for C88H162N6O2 $[M+2H]^{2+}$= 668.6452, found = 668.6450.

PPZ-B10: colorless oil, yield 47%. ^1H NMR (500 MHz, $CDCl_3$) δ 7.22 (t, *J* = 5.4 Hz, 2H), 3.30 (q, *J* = 6.2 Hz, 4H), 2.81–2.25 (m, 24H), 2.19 (t, *J* = 7.1 Hz, 4H), 1.77 (p, *J* = 7.3 Hz, 3H), 1.66 (p, *J* = 6.5 Hz, 4H), 1.49 – 1.33 (m, 8H), 1.34 – 1.17 (m, 56H), 0.87 (t, *J* = 6.9 Hz, 13H). ^{13}C NMR (125 MHz, $CDCl_3$) δ 172.77, 57.23, 53.90, 53.48, 53.43, 39.06, 34.91, 31.92, 29.69, 29.64, 29.61, 29.35, 27.60, 26.62, 25.68, 22.88, 22.69, 14.13. HRMS (ESI) m/z calcd for C58H118N6O2 $[M+2H]^{2+}$= 466.4731, found = 466.4730.

PPZ-B11: colorless oil, yield 44%. ^1H NMR (500 MHz, $CDCl_3$) δ 7.27 (t, *J* = 5.5 Hz, 2H), 3.29 (q, *J* = 6.1 Hz, 4H), 2.63 – 2.35 (m, 24H), 2.21 (t, *J* = 7.1 Hz, 4H), 1.80 (p, *J* = 7.1 Hz, 4H), 1.66 (p, *J* = 6.5 Hz, 4H), 1.50 – 1.40 (m, 8H), 1.34-1.16 (m, 64H), 0.86 (t, *J* = 6.9 Hz, 11H). ^{13}C NMR (125 MHz, CDCl3) δ 172.59, 57.17, 53.74, 53.39, 53.33, 39.05, 34.67, 31.92, 29.66, 29.63, 29.57, 29.35, 27.52, 26.23, 25.63, 22.69, 22.54, 14.13. HRMS (ESI) m/z calcd for C62H126N6O2 $[M+2H]^{2+}$= 494.5044, found = 494.5044.

PPZ-B12: colorless oil, yield 42%. ^1H NMR (500 MHz, $CDCl_3$) δ 7.26 (t, *J* = 5.7 Hz, 2H), 3.28 (q, *J* = 6.2 Hz, 4H), 2.59 – 2.30 (m, 24H), 2.19 (t, *J* = 7.1 Hz, 4H), 1.78 (p, *J* = 7.1 Hz, 4H), 1.64 (p, *J* = 6.5 Hz, 4H), 1.47 – 1.35 (m, 8H), 1.30 – 1.15 (m, 72H), 0.85 (t, *J* = 6.9 Hz, 12H). ^{13}C NMR (125 MHz, $CDCl_3$) δ 172.62, 57.17, 53.77, 53.39, 53.35, 39.03, 34.70, 31.91, 29.67, 29.66, 29.64, 29.58, 29.35, 27.53, 26.32, 25.64, 22.68, 22.61, 14.12. HRMS (ESI) m/z calcd for C66H134N6O2 $[M+2H]^{2+}$= 522.5357, found = 522.5351.

PPZ-B18-2Z: colorless oil, yield 32%. ^1H NMR (500 MHz, $CDCl_3$) δ 7.50 (t, *J* = 5.5 Hz, 1H), 5.41 – 5.25 (m, 16H), 3.27 (q, *J* = 6.1 Hz, 4H), 2.82 – 2.40 (m, 32H), 2.30 (t, *J* = 6.9 Hz, 4H), 2.02 (q, *J* = 6.9 Hz, 14H), 1.93 (p, *J* = 7.0 Hz, 4H), 1.67 (p, *J* = 6.6 Hz, 4H), 1.61 – 1.53 (m, 8H), 1.39 – 1.18 (m, 66H), 0.86 (t, *J* = 6.9 Hz, 12H). ^{13}C NMR (125 MHz, $CDCl_3$) δ 171.94, 56.88, 53.15, 53.10, 52.78, 38.92, 31.51, 29.62, 29.47, 29.34, 29.32, 29.20, 27.21, 27.19, 25.63, 25.45, 22.57, 14.09. HRMS (ESI) m/z calcd for C90H166N6O2 $[M+2H]^{2+}$= 682.6609, found = 682.6608.

B. Supplementary Figures

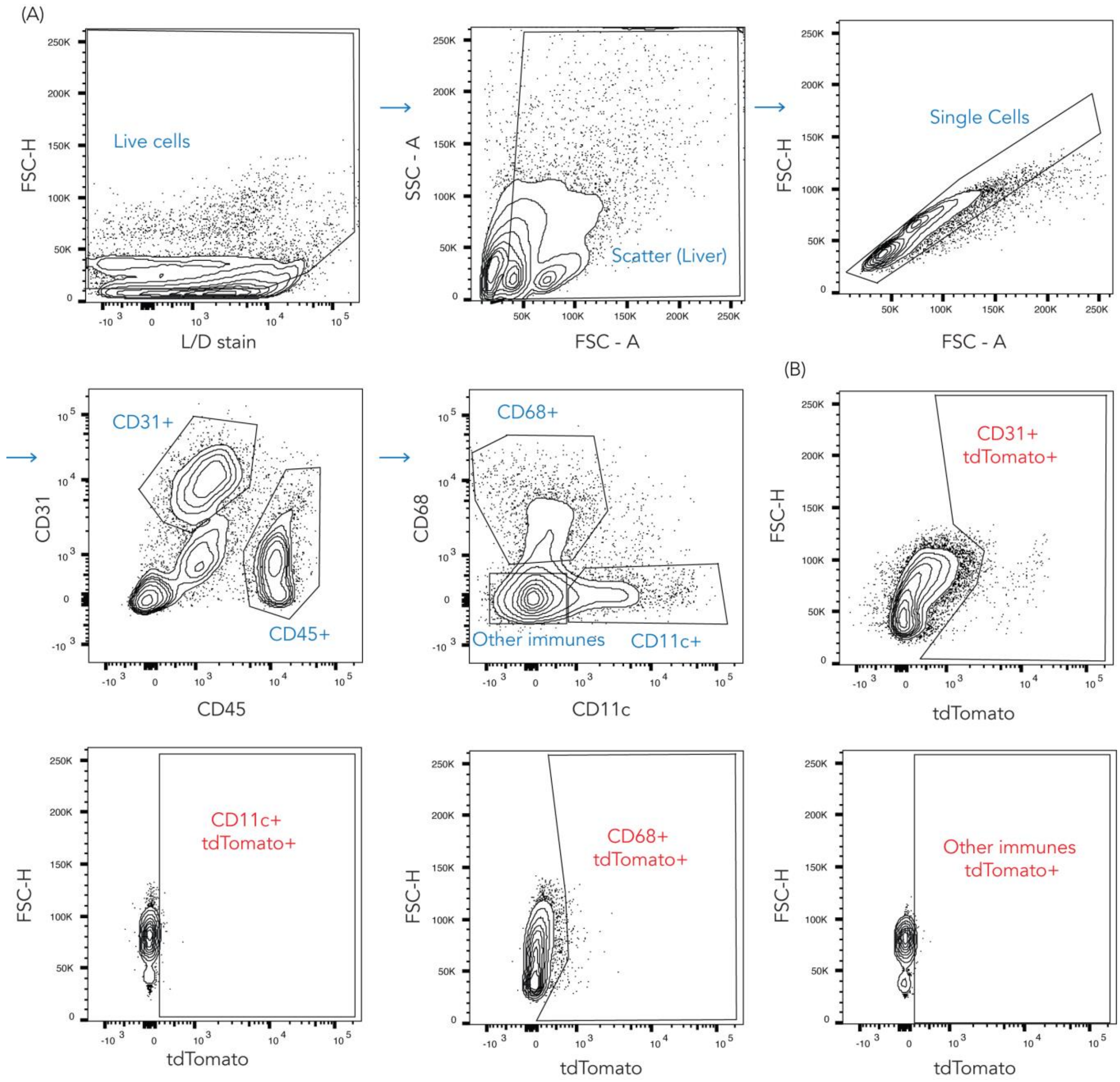

Supplementary Figure 1. *Representative gating strategies for FACS for cell types in the liver.*

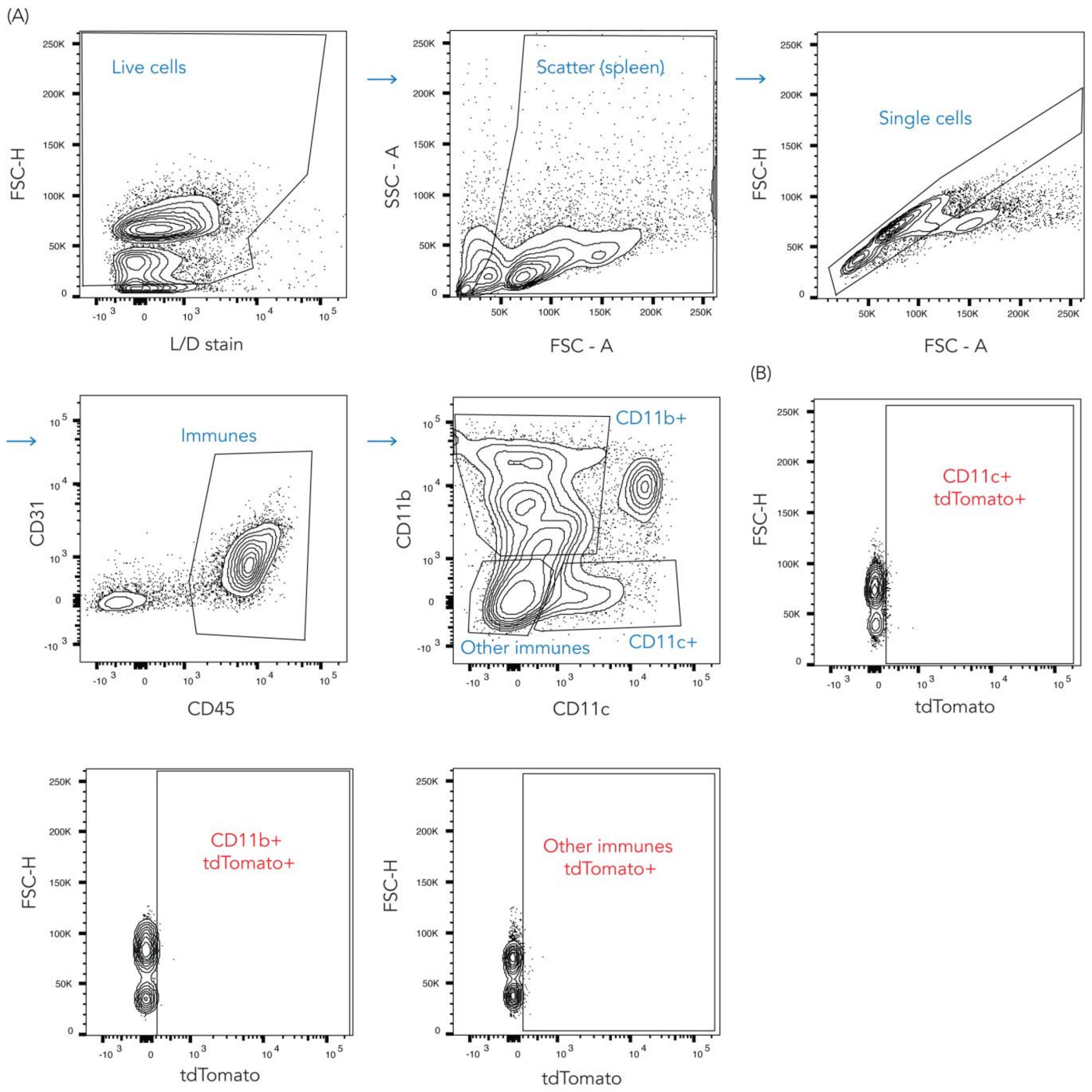

Supplementary Figure 2. *Representative gating strategies for FACS for cell types in the spleen.*

		Raw Counts Cell type X (%)			
LNP	Barcode	Mouse 1	Mouse 2	Mouse 3	Input
1	GACACAGT	100	80	200	100
2	GCATAACG	50	45	110	120
3	ACAGAGGT	120	105	250	110
Total Counts		270	230	560	330

		Normalized Counts Cell type X (%)			
LNP	Barcode	Mouse 1	Mouse 2	Mouse 3	Input
1	GACACAGT	37	35	36	30
2	GCATAACG	19	20	20	36
3	ACAGAGGT	44	46	45	33
Total Counts		100	100	100	100

		Normalized to Input Cell type X (%)		
LNP	Barcode	Mouse 1	Mouse 2	Mouse 3
1	GACACAGT	40	38	39
2	GCATAACG	17	18	18
3	ACAGAGGT	44	45	44
Total Counts		100	100	100

Supplementary Figure 3. *Normalized delivery example calculation. In the first step, the total barcode counts in a given sample are summed. In the second step, the normalized counts for each barcode are calculated as Barcode 1 / Sum (Barcode 1-->N). In the third step, these normalized counts are normalized a second time by the input DNA. Data from the third step are then plotted as normalized delivery.*

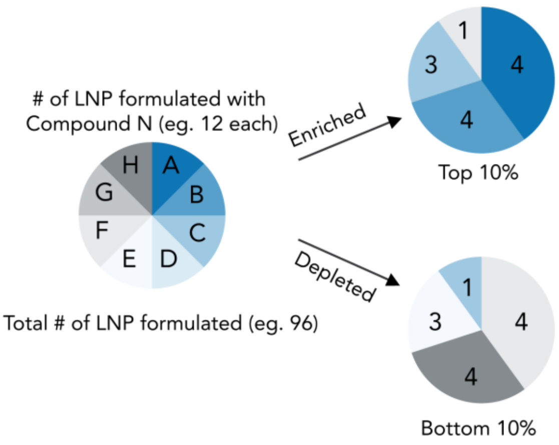

$$\frac{\text{\# of compound N in top/bottom 10\%}}{\text{total \# of LNP X 10\%}} \div \frac{\text{\# of compound N formulated in total}}{\text{total \# of LNP}} = \text{Enriched/ depletd}$$

Fold of enrichment of compound N = Compound N enriched - compound N depleted

eg. For compound A as an example, enriched = 4/10 ÷ 12/96 = 3.2; depleted = 0/10 ÷ 12/96; Fold of enrichment = 3.2

Supplementary Figure 4. *Enrichment calculation example.*

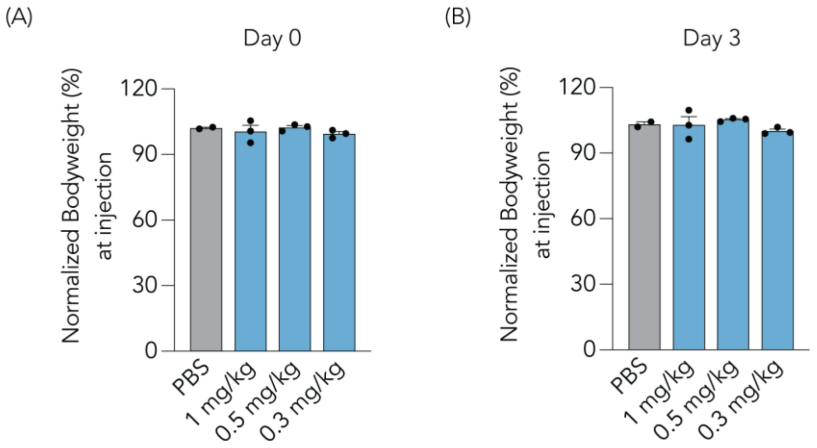

Supplementary Figure 5. *(A) Mouse weights 24 hrs after administration of PBS or* ***LNP-A10*** *at different doses of Cre mRNA. Average +/- SEM, N = 2-3/group. (B) Mouse weights 3 days after administration of PBS or* ***LNP-A10*** *at different doses of Cre mRNA. Average +/- SEM, N = 2-3/group.*

C. References

1. Chen, D.; Love, K. T. Chen, Y. Eltoukhy, A. A. Kastrup, C. Sahay, G. Jeon, A. Dong, Y. Whitehead, K. A. Anderson, D. G. *J Am Chem Soc* **2012**, *134*, 6948.

2. Sago, C. D. *et al.*, Modifying a Commonly Expressed Endocytic Receptor Retargets Nanoparticles in Vivo. *Nano letters*, **2018**, *18*, 7590.

3. Geall, A. J. et al. Nonviral delivery of self-amplifying RNA vaccines, *PNAS*, **2012**, *109*, 14604.

4.Dahlman, J. E.; Kauffman, K. J.; Xing, Y.; Shaw, T. E.; Mir, F. F.; Dlott, C. C.; Langer, R.; Anderson, D. G.; Wang, E. T. *PNAS*, **2017**, *114*, 2060.

D. NMR Spectra of Intermediates and Ionizable Lipids

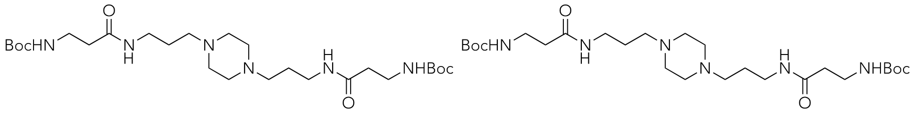

Intermediate 4

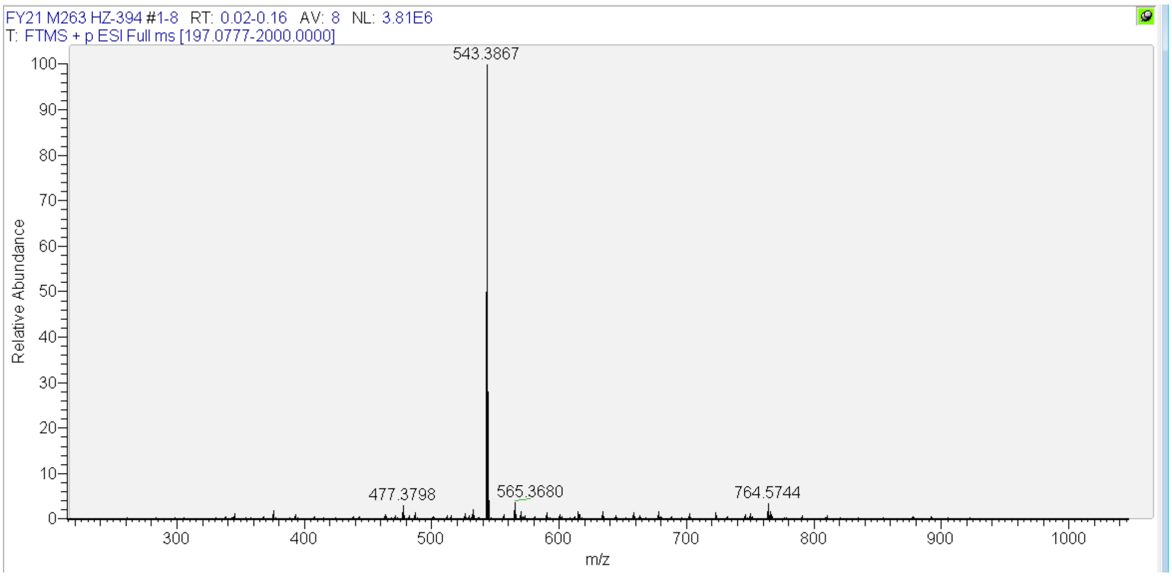

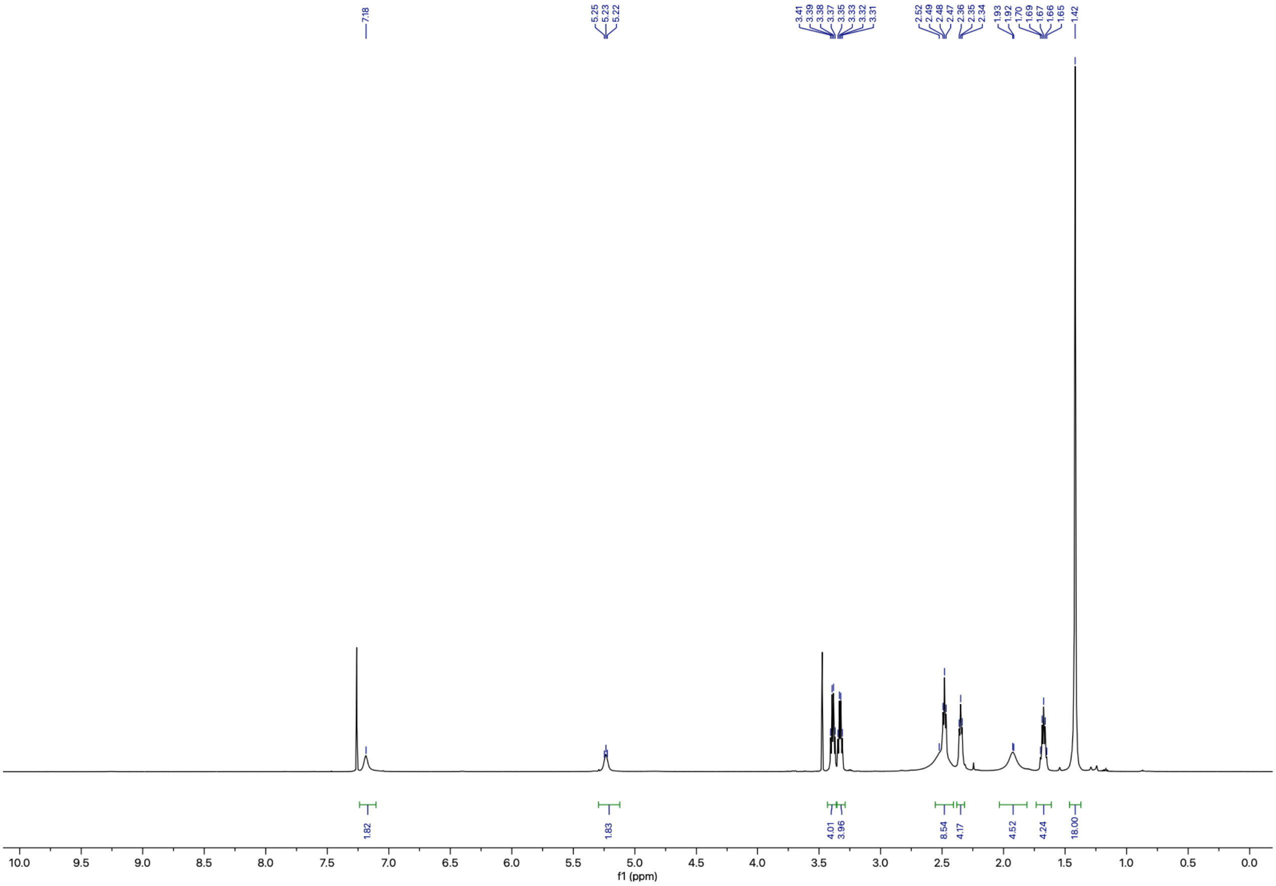

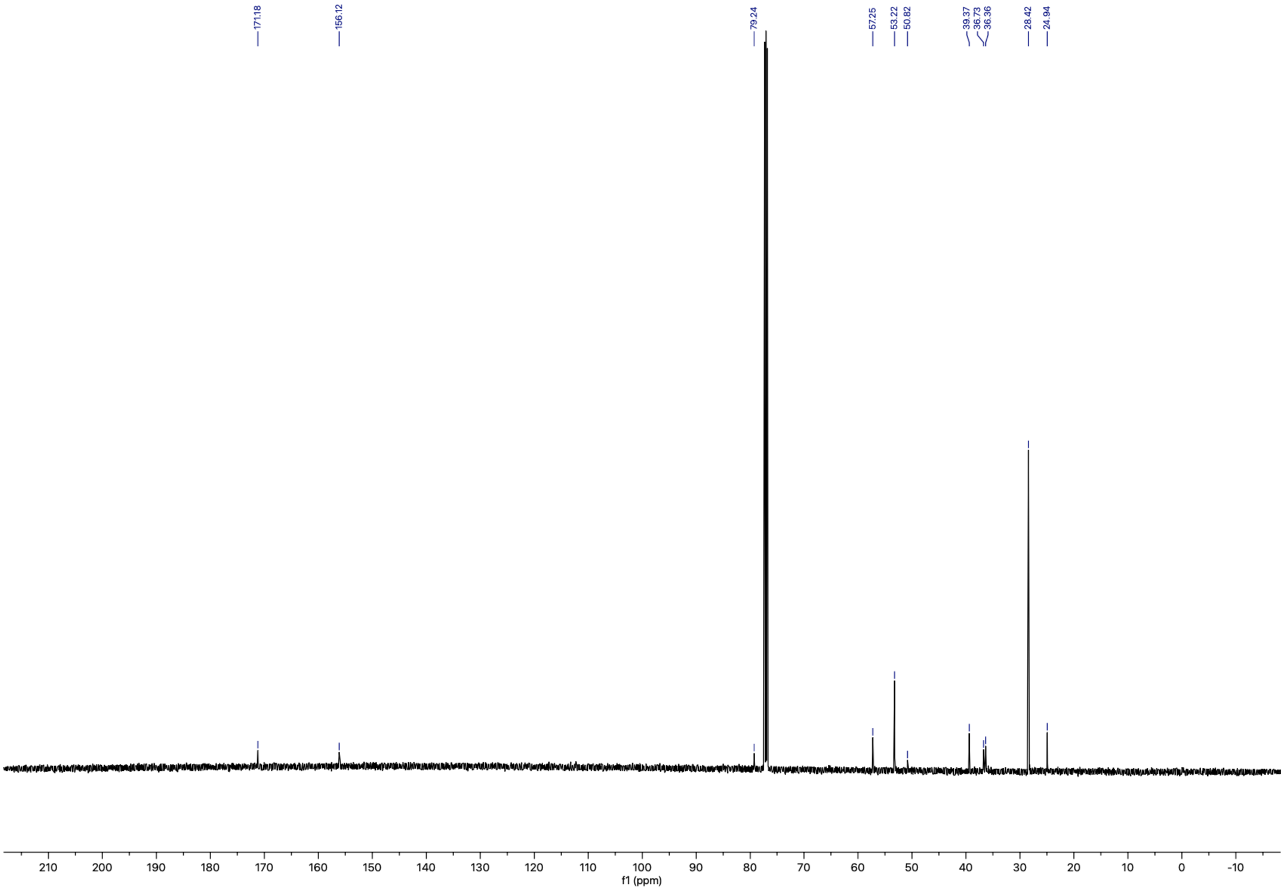

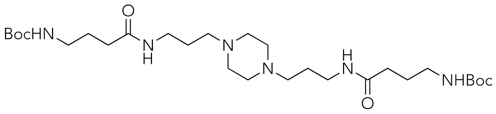

Intermediate 5

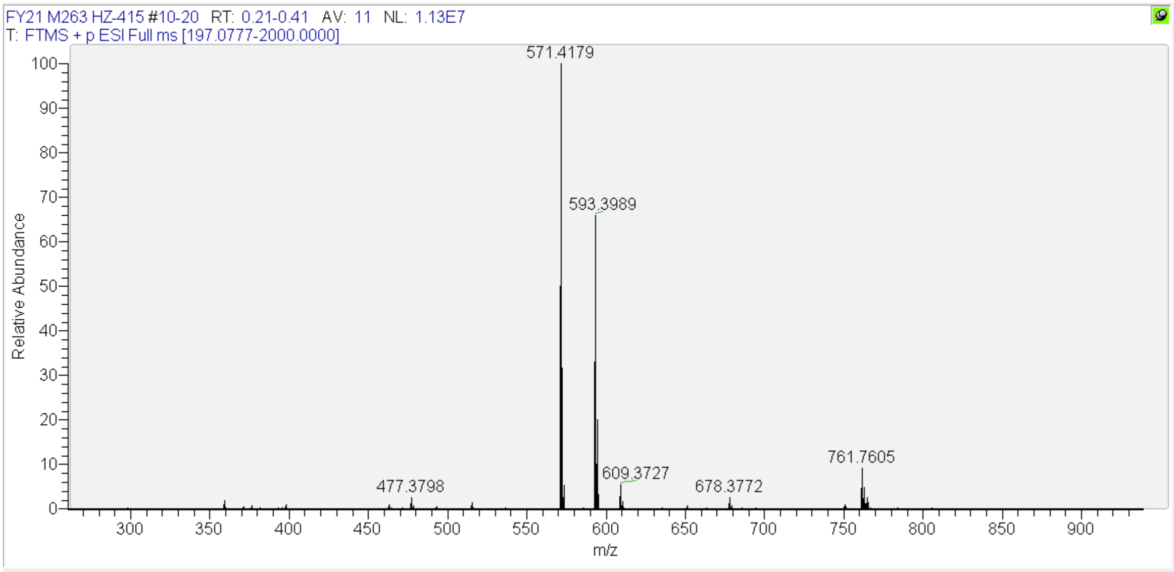

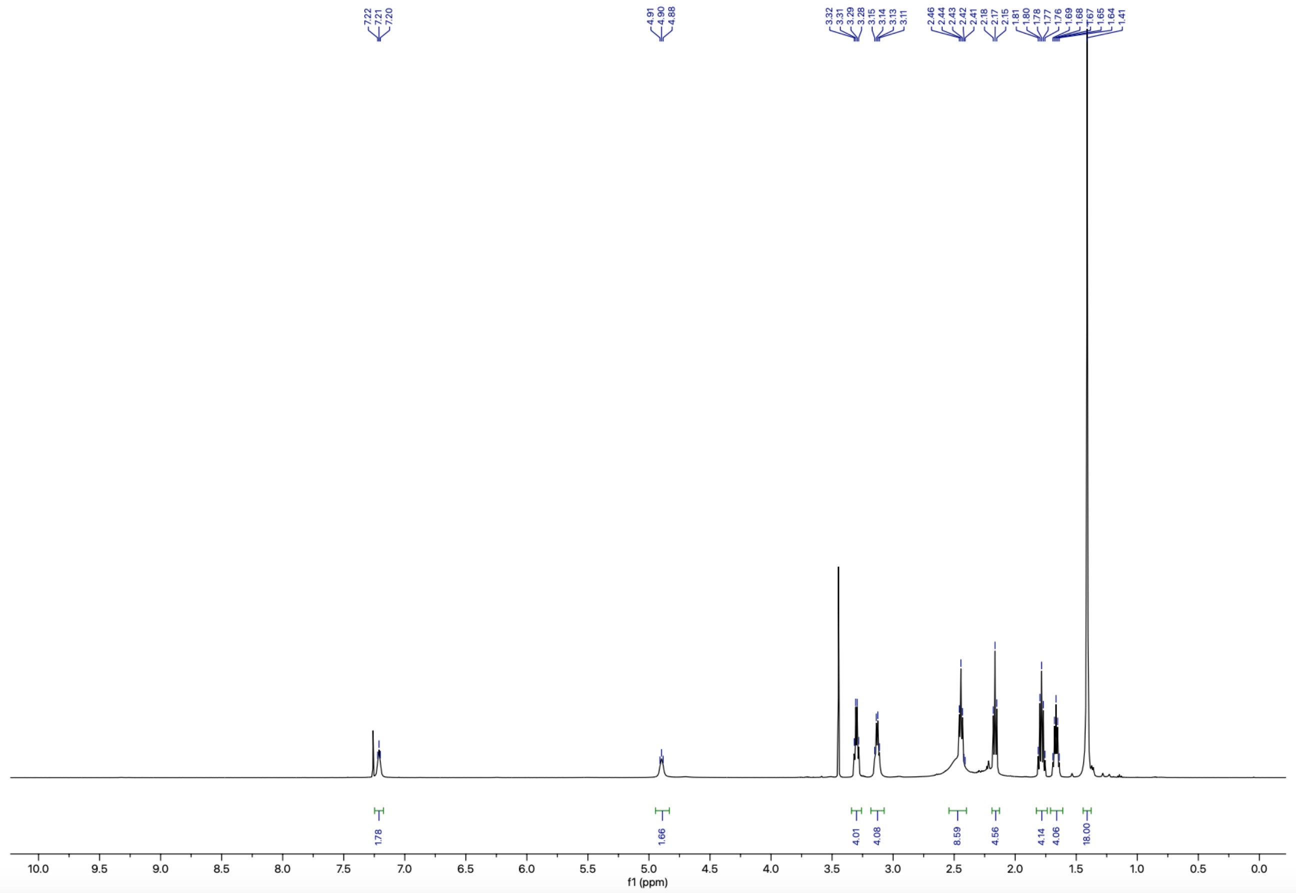
7.22
7.21
7.20
4.91
4.90
4.88
3.32
3.31
3.29
3.28
3.15
3.14
3.13
3.11
2.46
2.44
2.43
2.42
2.41
2.18
2.17
2.15
1.81
1.80
1.78
1.77
1.76
1.69
1.68
1.67
1.65
1.64
1.41
1.78
1.66
4.01
4.08
8.59
4.56
4.14
4.06
18.00
f1 (ppm)

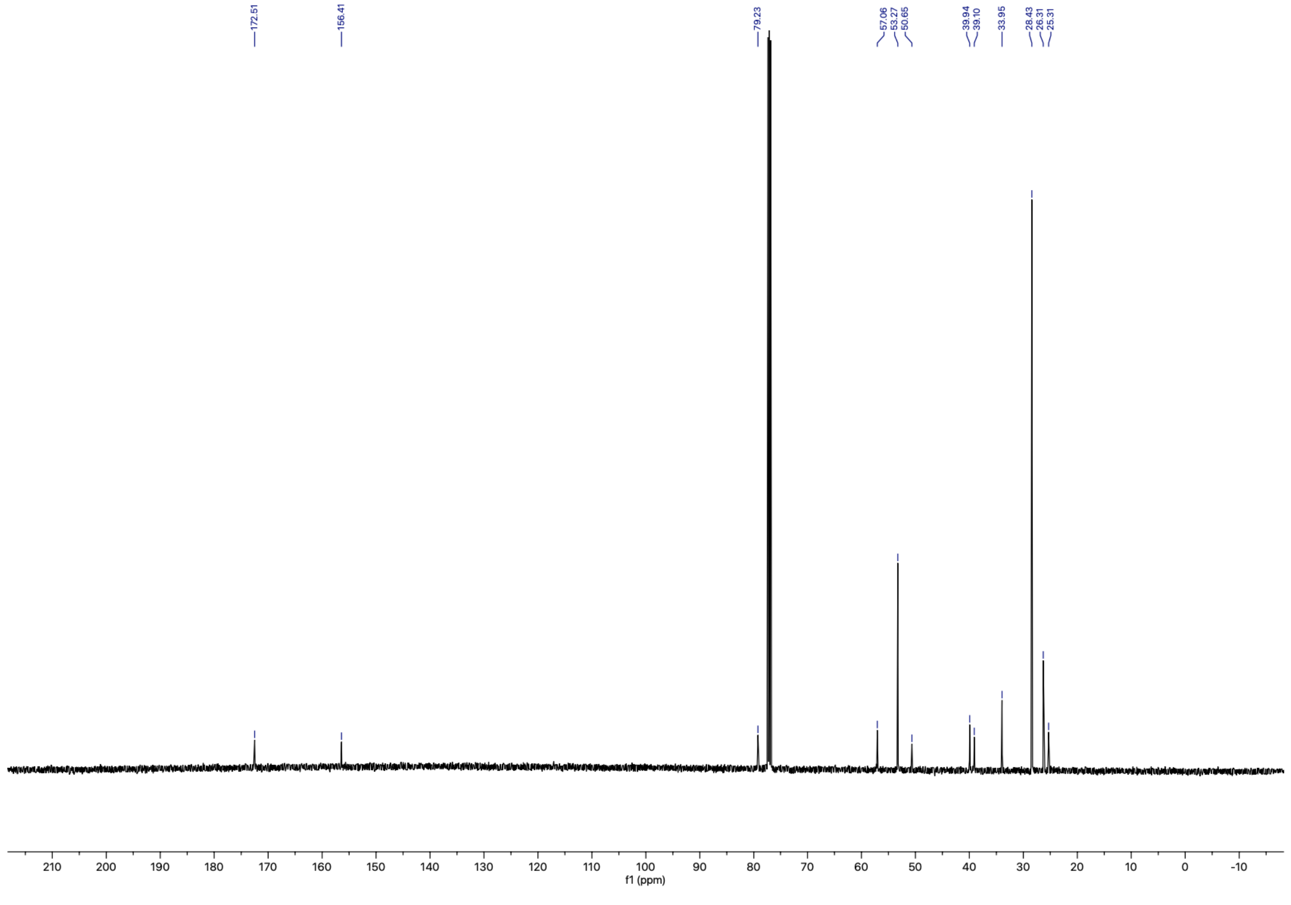
172.51
156.41
79.23
57.06
53.27
50.65
39.94
39.10
33.95
28.43
26.31
25.31
f1 (ppm)

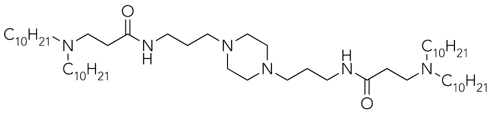
O
$C_{10}H_{21}$
N
H
N
N
N
H
N
O
$C_{10}H_{21}$
$C_{10}H_{21}$
N
$C_{10}H_{21}$

PPZ-A10

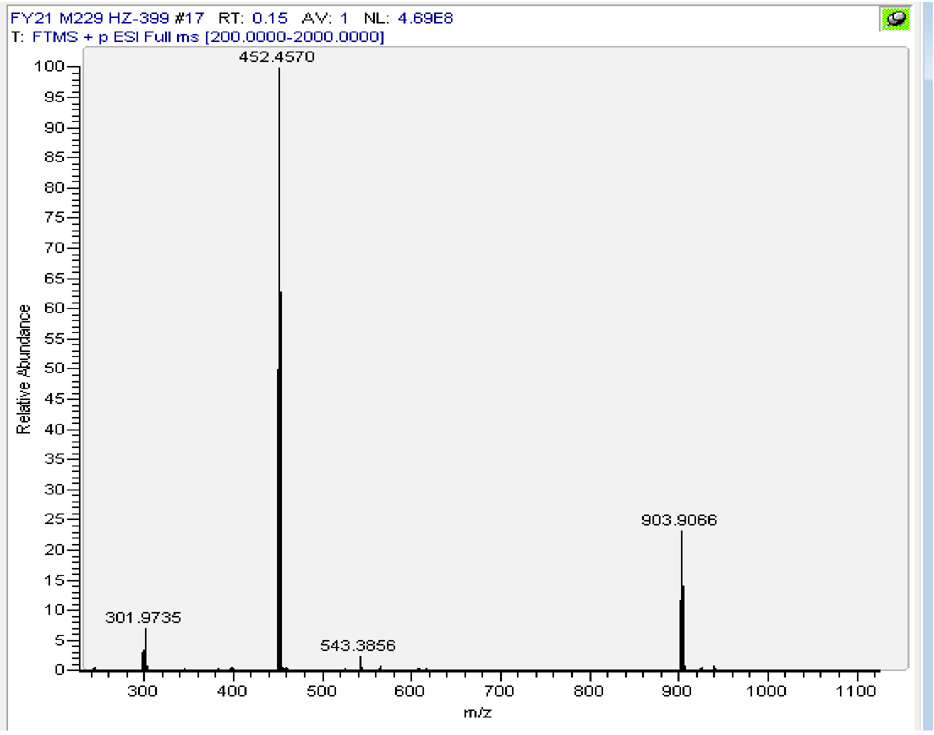
FY21 M229 HZ-399 #17 RT: 0.15 AV: 1 NL: 4.69E8
T: FTMS + p ESI Full ms [200.0000-2000.0000]
452.4570
903.9066
301.9735
543.3856
Relative Abundance
m/z

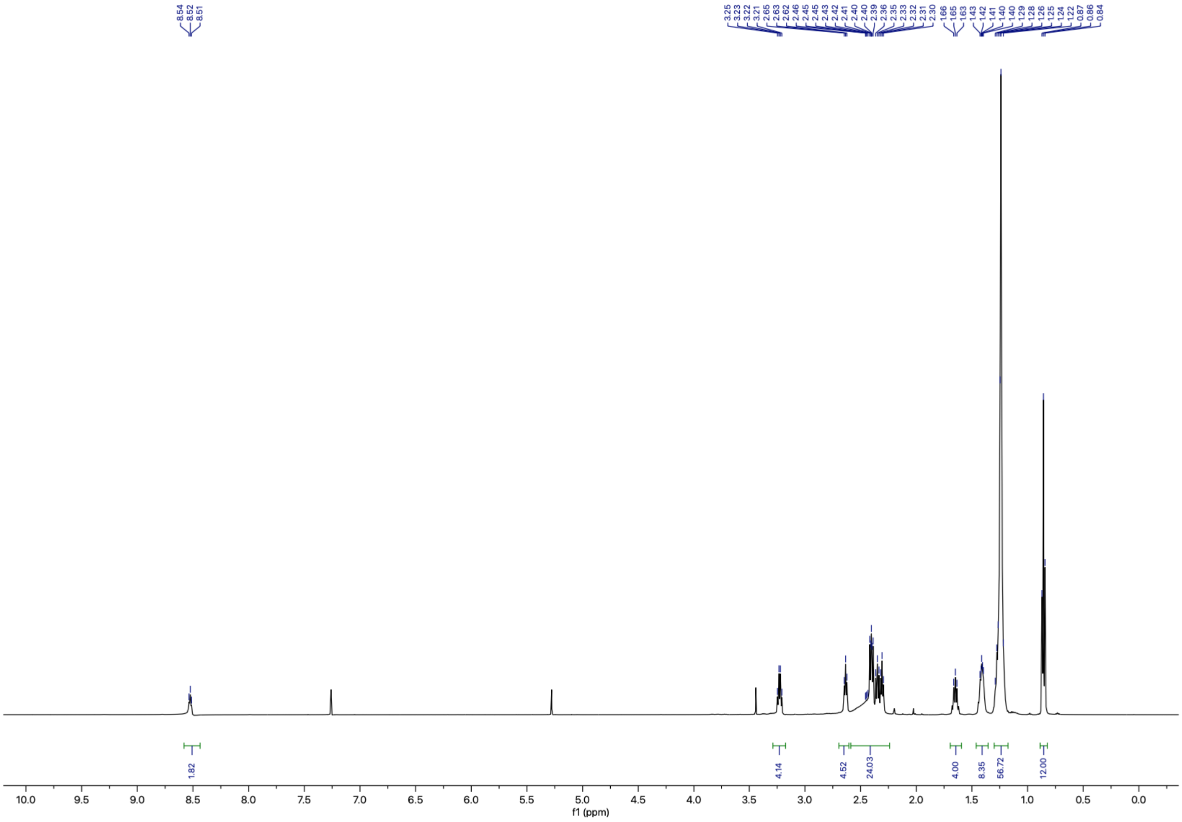
1.82
4.14
4.52
24.03
4.00
8.35
56.72
12.00
f1 (ppm)

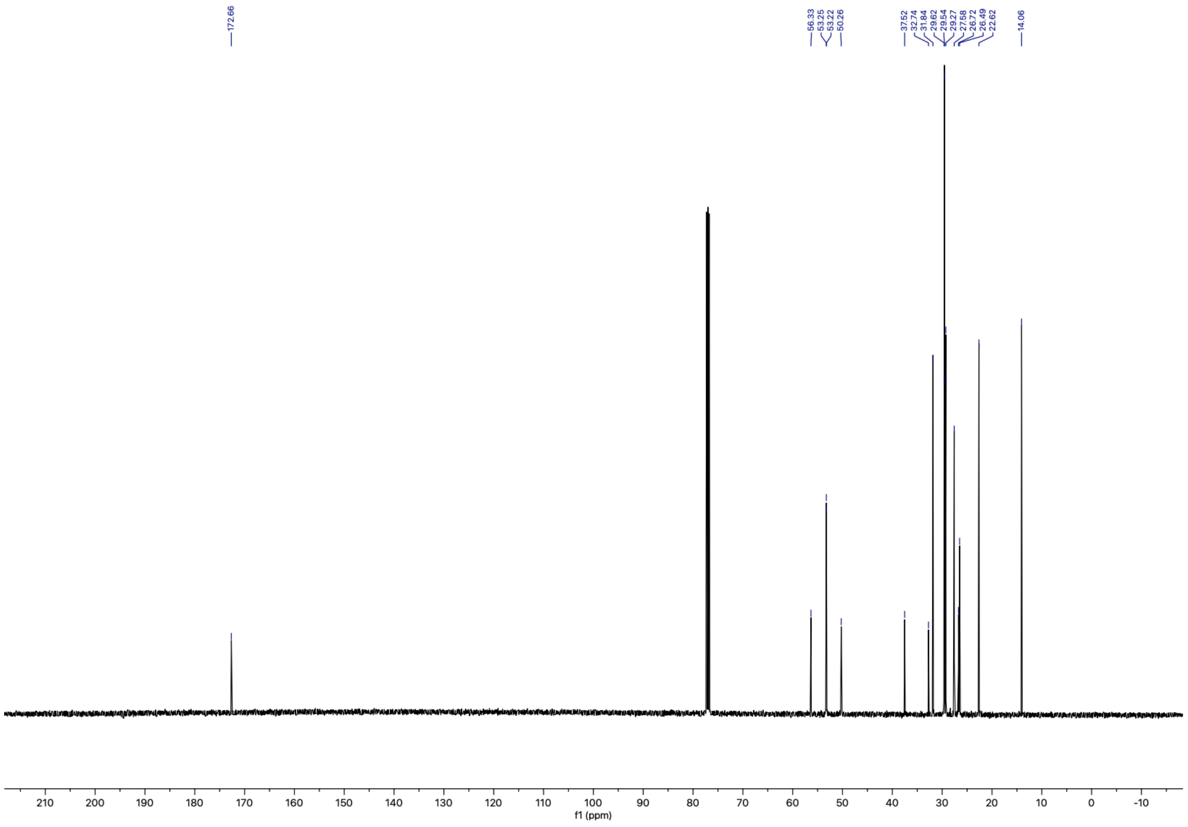
172.66
56.33
53.25
53.22
50.26
37.52
32.74
31.84
29.62
29.54
29.27
27.58
26.72
26.49
22.62
14.06
210 200 190 180 170 160 150 140 130 120 110 100 90 80 70 60 50 40 30 20 10 0 -10
f1 (ppm)

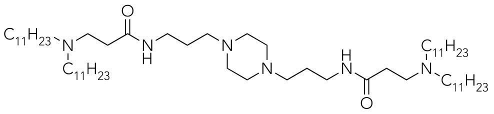

PPZ-A11

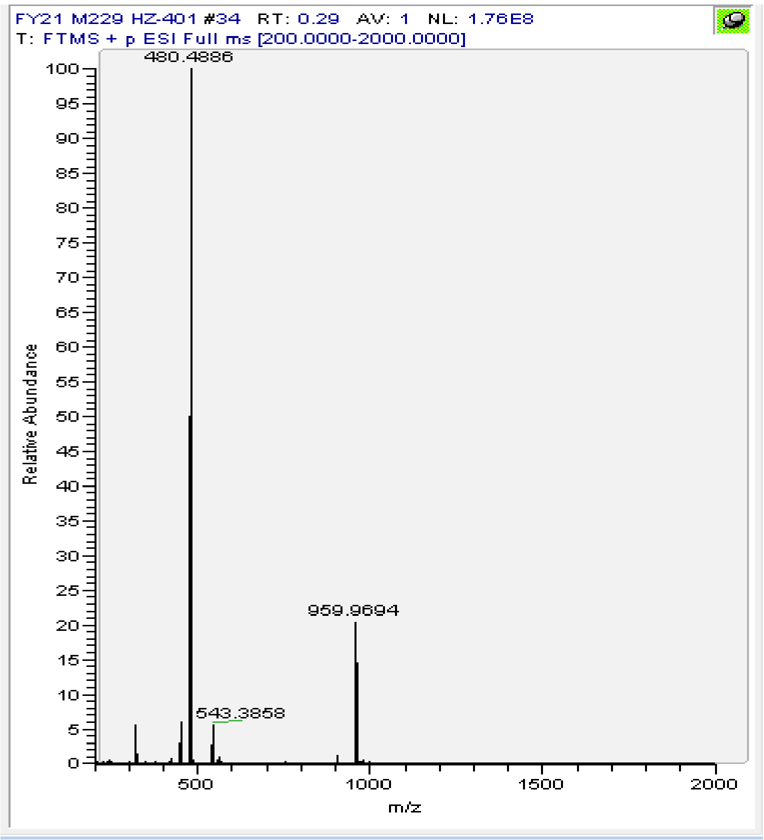
FY21 M229 HZ-401 #34 RT: 0.29 AV: 1 NL: 1.76E8
T: FTMS + p ESI Full ms [200.0000-2000.0000]
480.4886
959.9694
543.3858
Relative Abundance
m/z

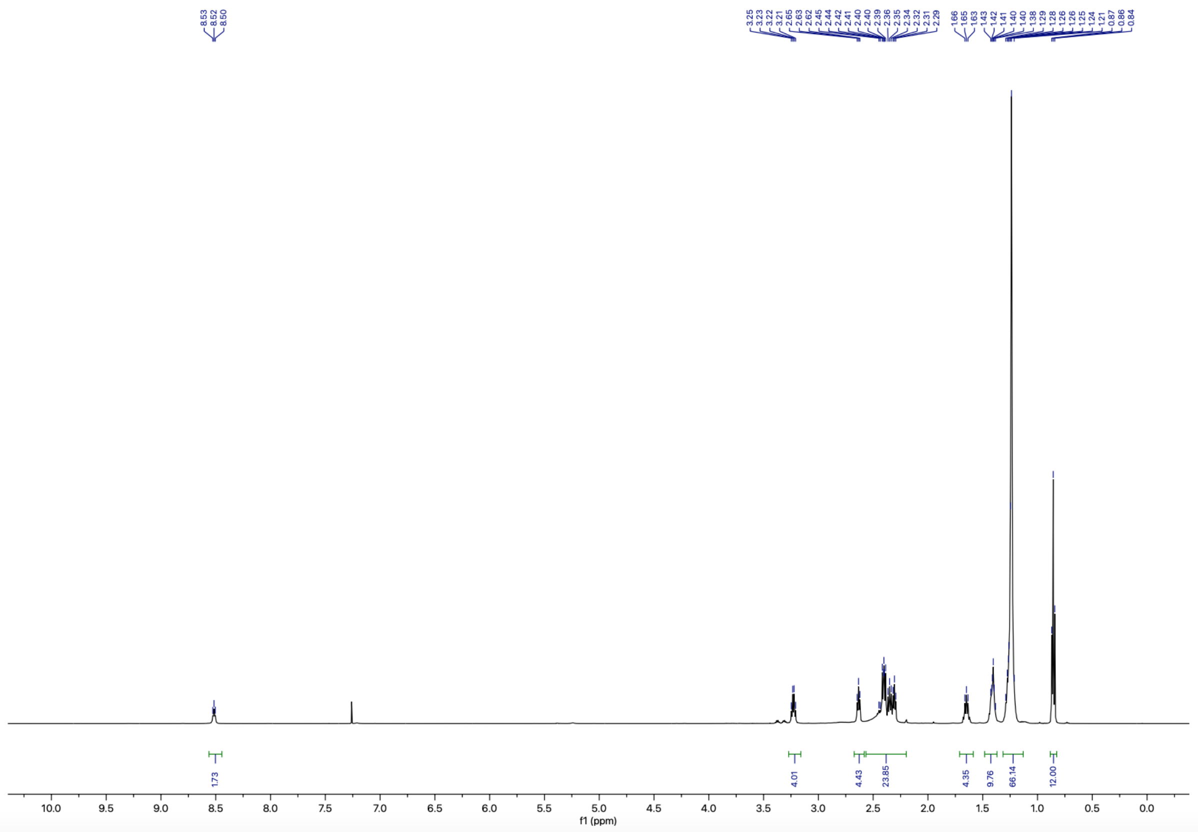
8.53
8.52
8.50
3.25
3.23
3.22
3.21
2.65
2.63
2.62
2.45
2.44
2.42
2.41
2.40
2.40
2.39
2.36
2.35
2.34
2.32
2.31
2.29
1.66
1.65
1.63
1.43
1.42
1.41
1.40
1.40
1.38
1.29
1.28
1.26
1.26
1.25
1.24
1.21
0.87
0.86
0.84
1.73
4.01
4.43
23.85
4.35
9.76
66.14
12.00
10.0 9.5 9.0 8.5 8.0 7.5 7.0 6.5 6.0 5.5 5.0 4.5 4.0 3.5 3.0 2.5 2.0 1.5 1.0 0.5 0.0
f1 (ppm)

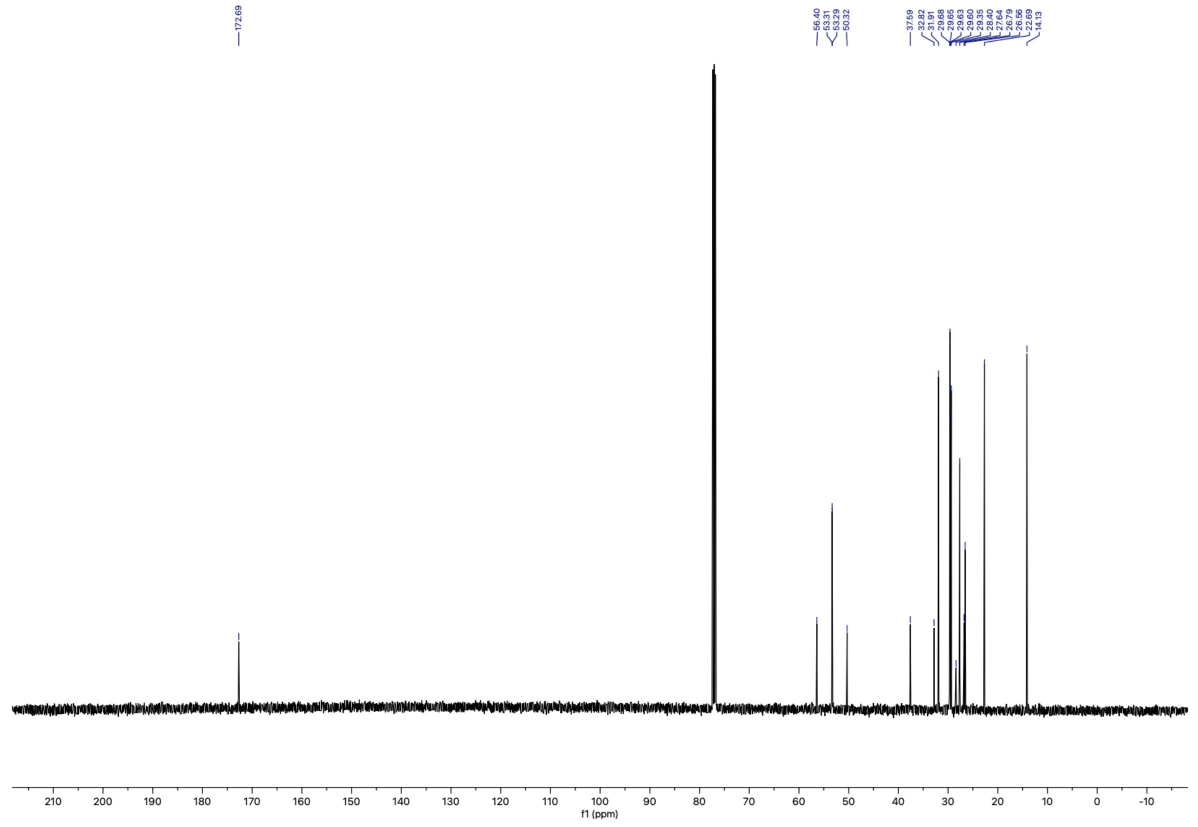
172.69
56.40
53.31
53.29
50.32
37.59
32.82
31.91
29.68
29.65
29.63
29.60
29.35
28.40
27.64
26.79
26.56
22.69
14.13
210 200 190 180 170 160 150 140 130 120 110 100 90 80 70 60 50 40 30 20 10 0 -10
f1 (ppm)

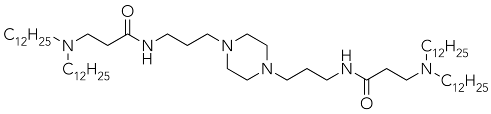
C12H25
N
C12H25
O
N
H
N
N
H
N
O
C12H25
N
C12H25

PPZ-A12

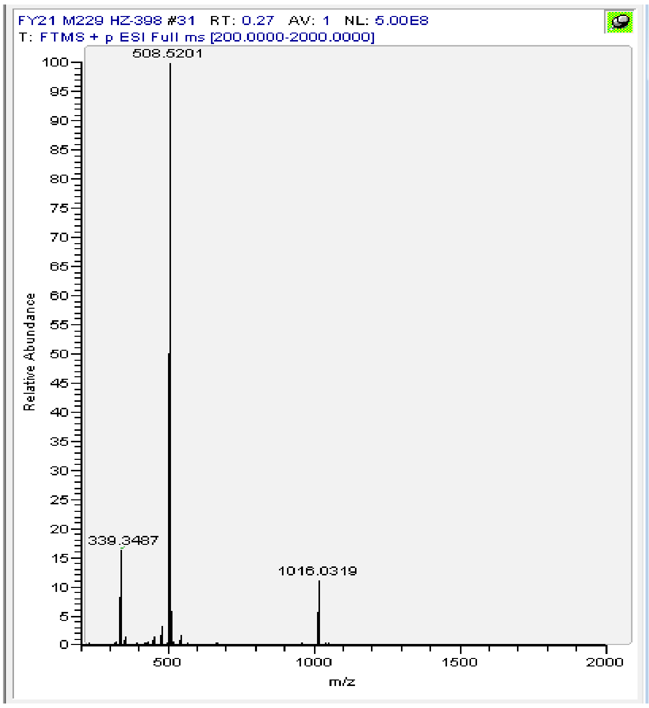
FY21 M229 HZ-398 #31 RT: 0.27 AV: 1 NL: 5.00E8
T: FTMS + p ESI Full ms [200.0000-2000.0000]
508.5201
339.3487
1016.0319
Relative Abundance
m/z

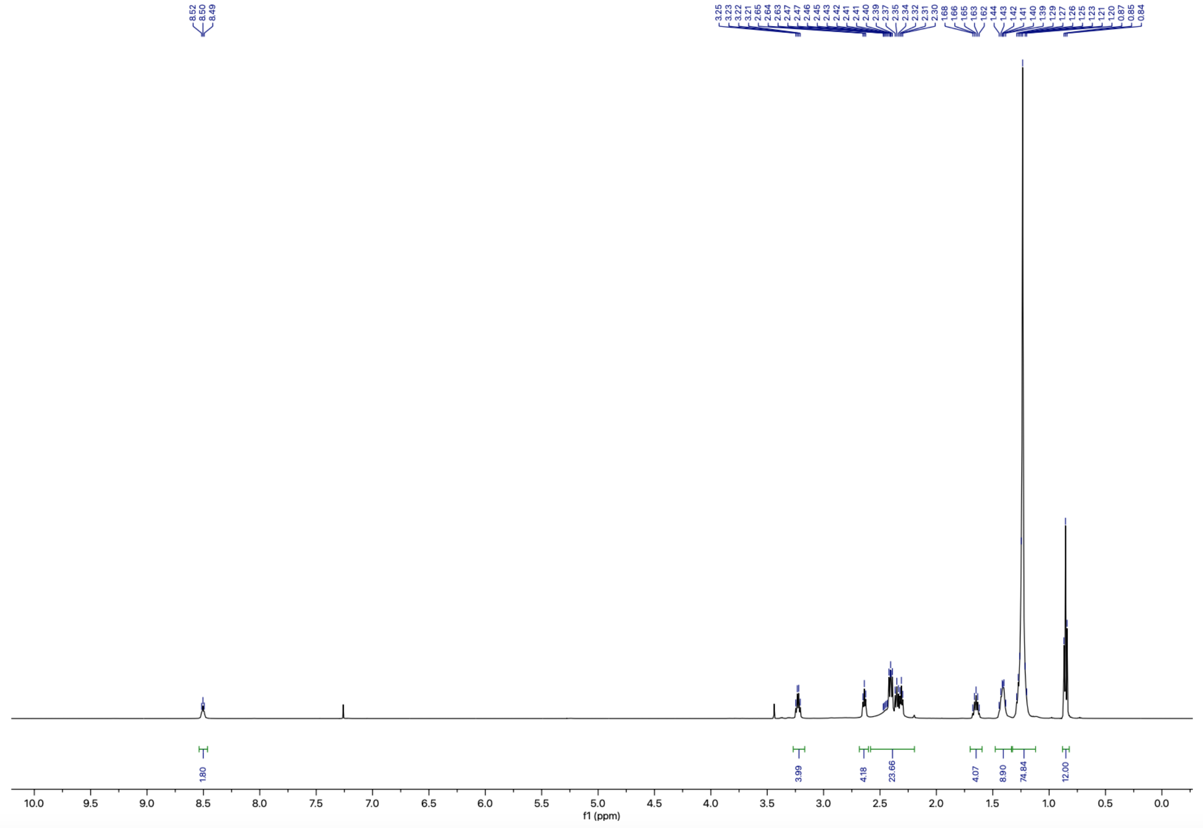
8.52
8.50
8.49
1.80
3.99
4.18
23.66
4.07
8.90
74.84
12.00
f1 (ppm)

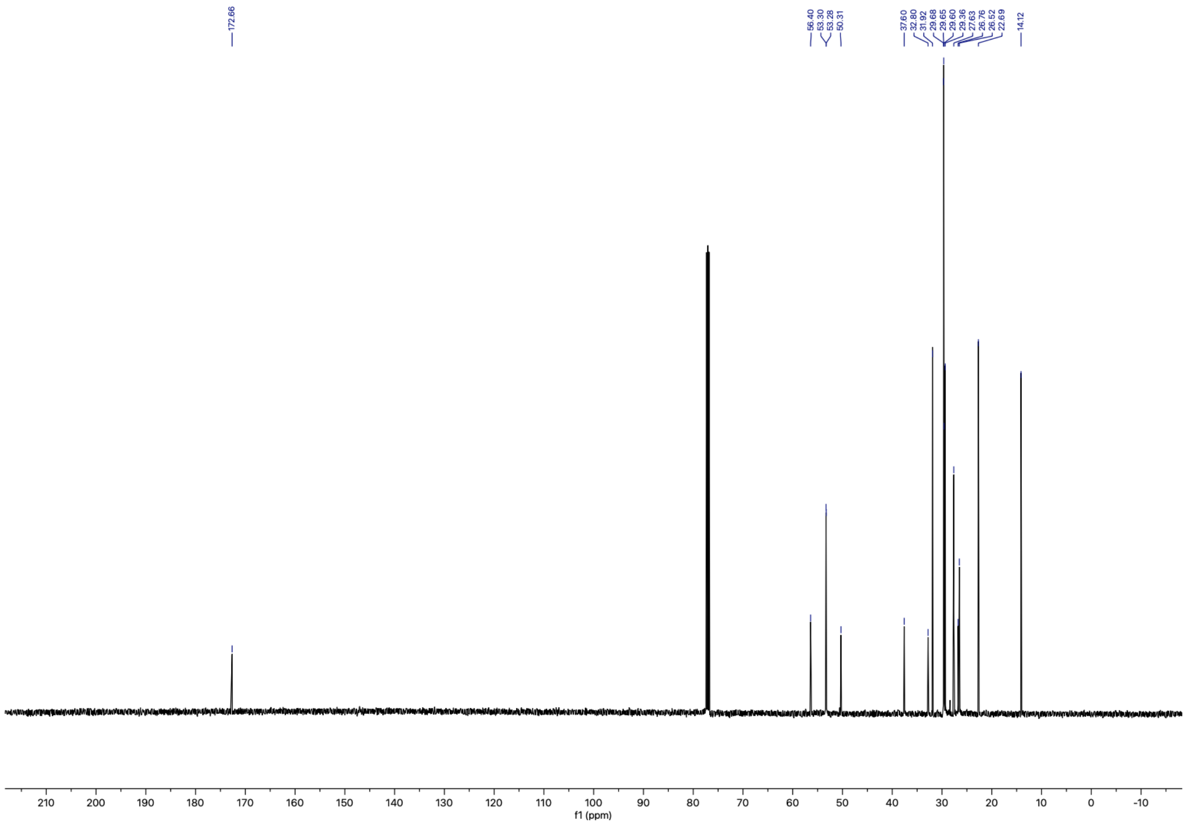
172.66
56.40
53.30
53.28
50.31
37.60
32.80
31.92
29.68
29.65
29.60
29.36
27.63
26.76
26.52
22.69
14.12
f1 (ppm)

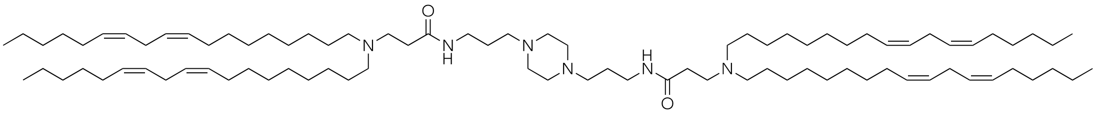

PPZ-A18-2Z

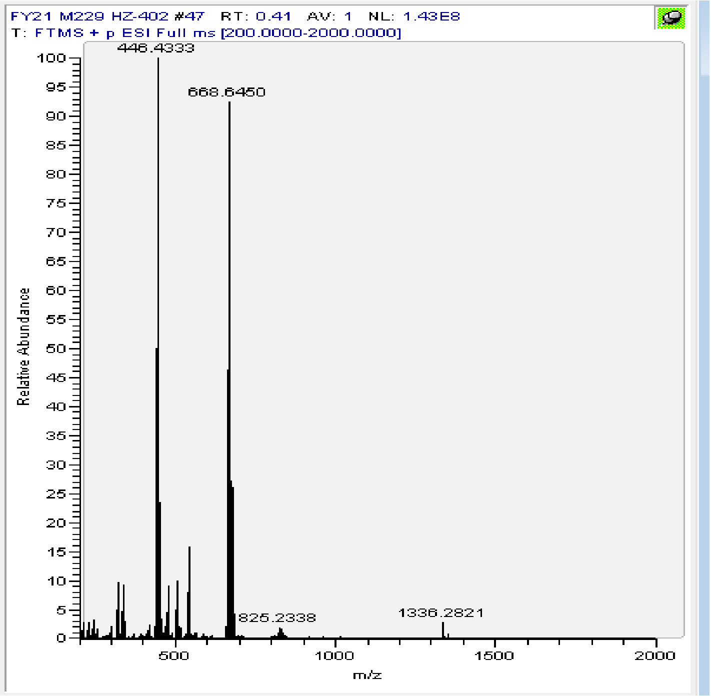
FY21 M229 HZ-402 #47 RT: 0.41 AV: 1 NL: 1.43E8
T: FTMS + p ESI Full ms [200.0000-2000.0000]
446.4333
668.6450
825.2338
1336.2821
Relative Abundance
m/z

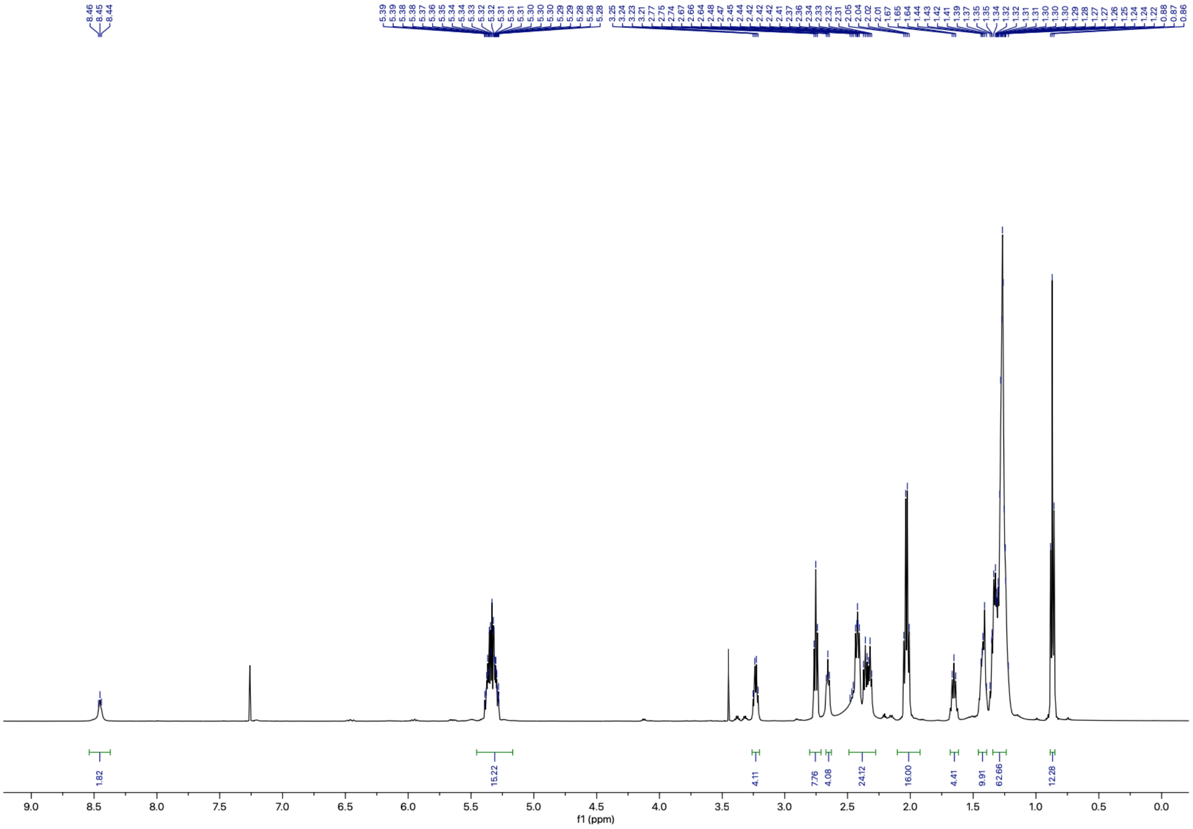
8.46
8.45
8.44
5.39 5.39 5.38 5.38 5.37 5.36 5.35 5.34 5.34 5.33 5.32 5.32 5.31 5.31 5.31 5.30 5.30 5.30 5.29 5.29 5.28 5.28 5.28
3.25 3.24 3.23 3.21 2.77 2.75 2.74 2.67 2.66 2.64 2.48 2.47 2.45 2.44 2.42 2.42 2.42 2.41 2.37 2.36 2.34 2.33 2.32 2.31 2.05 2.04 2.02 2.01 1.67 1.65 1.64 1.44 1.43 1.42 1.41 1.39 1.37 1.35 1.35 1.34 1.32 1.32 1.31 1.31 1.30 1.30 1.30 1.29 1.28 1.27 1.27 1.26 1.25 1.24 1.24 1.22 0.88 0.87 0.86
1.82
15.22
4.11
7.76
4.08
24.12
16.00
4.41
9.91
62.66
12.28
9.0 8.5 8.0 7.5 7.0 6.5 6.0 5.5 5.0 4.5 4.0 3.5 3.0 2.5 2.0 1.5 1.0 0.5 0.0
f1 (ppm)

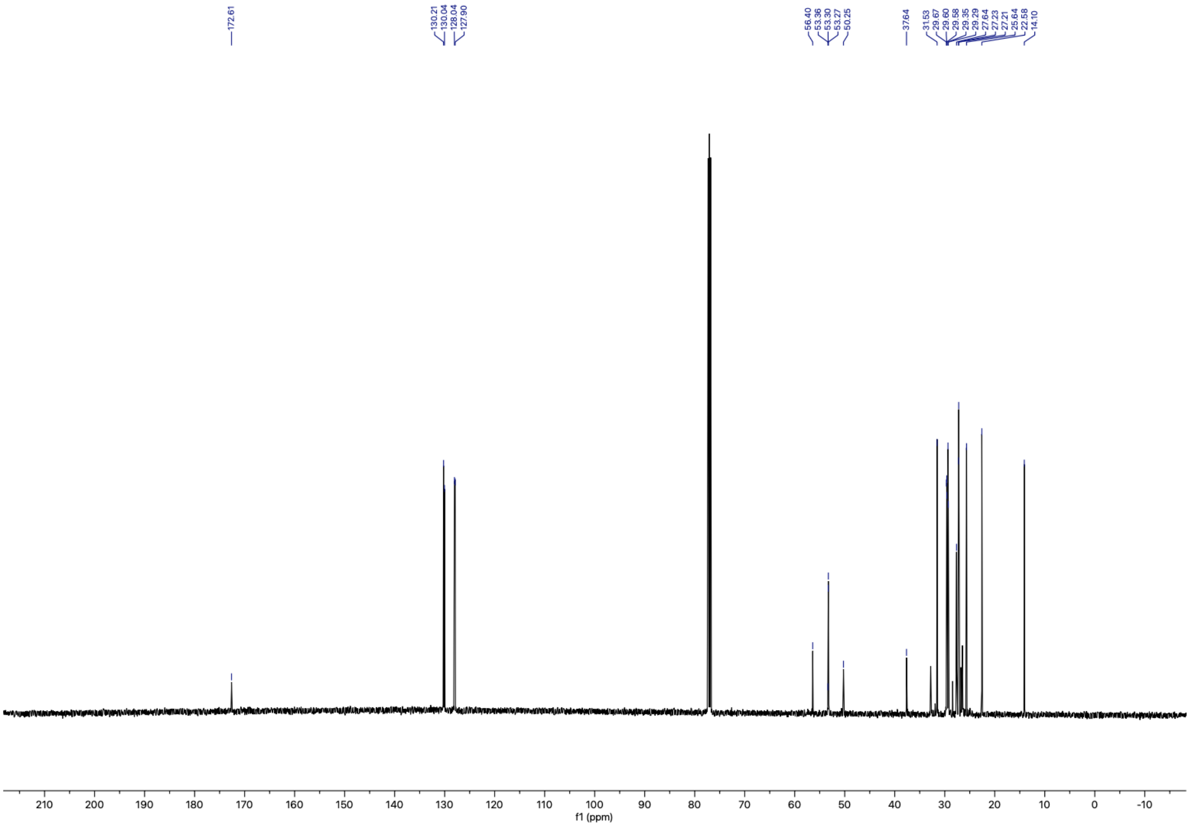
172.61
130.21 130.04 128.04 127.90
56.40 53.36 53.30 53.27 50.25
37.64
31.53 29.67 29.60 29.58 29.35 29.29 27.64 27.23 27.21 25.64 22.58 14.10
210 200 190 180 170 160 150 140 130 120 110 100 90 80 70 60 50 40 30 20 10 0 -10
f1 (ppm)

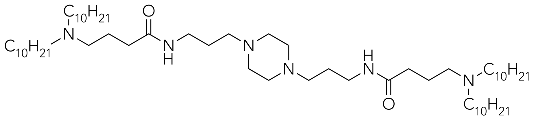

PPZ-B10

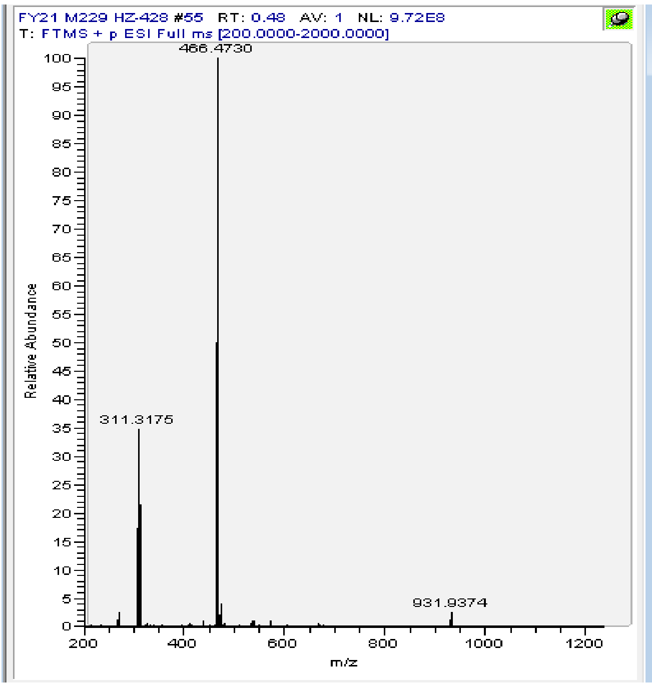

FY21 M229 HZ-428 #55 RT: 0.48 AV: 1 NL: 9.72E8
T: FTMS + p ESI Full ms [200.0000-2000.0000]
466.4730
311.3175
931.9374
Relative Abundance
m/z

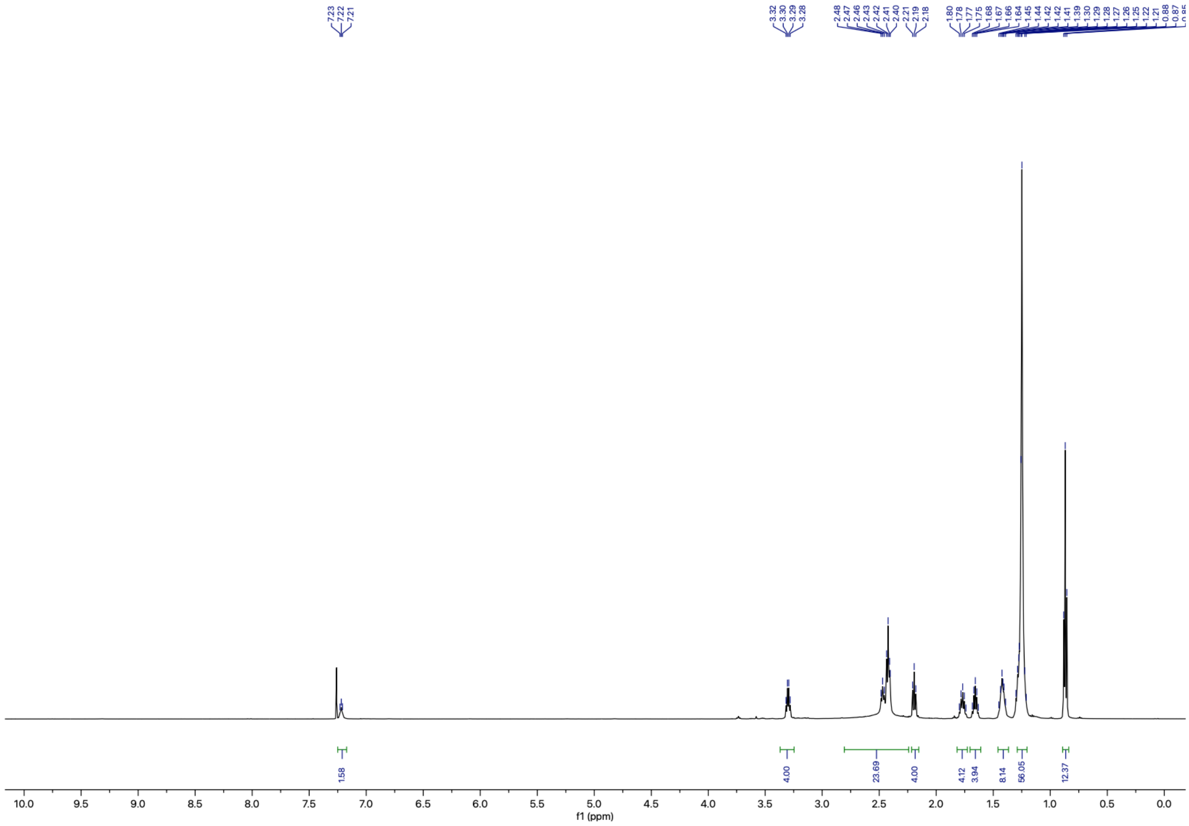

f1 (ppm)

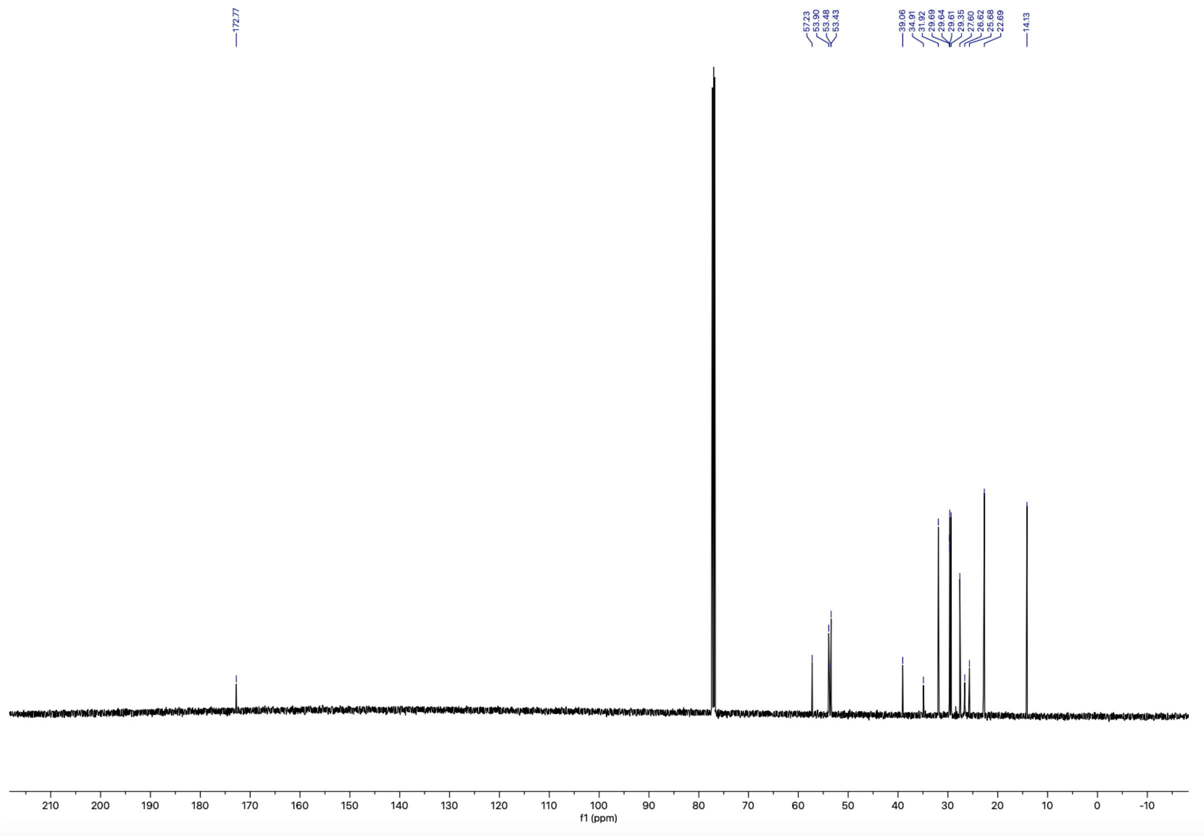
172.77
57.23
53.90
53.48
53.43
39.06
34.91
31.92
29.69
29.64
29.61
29.35
27.60
26.62
25.68
22.69
14.13
f1 (ppm)

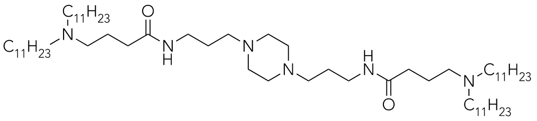

PPZ-B11

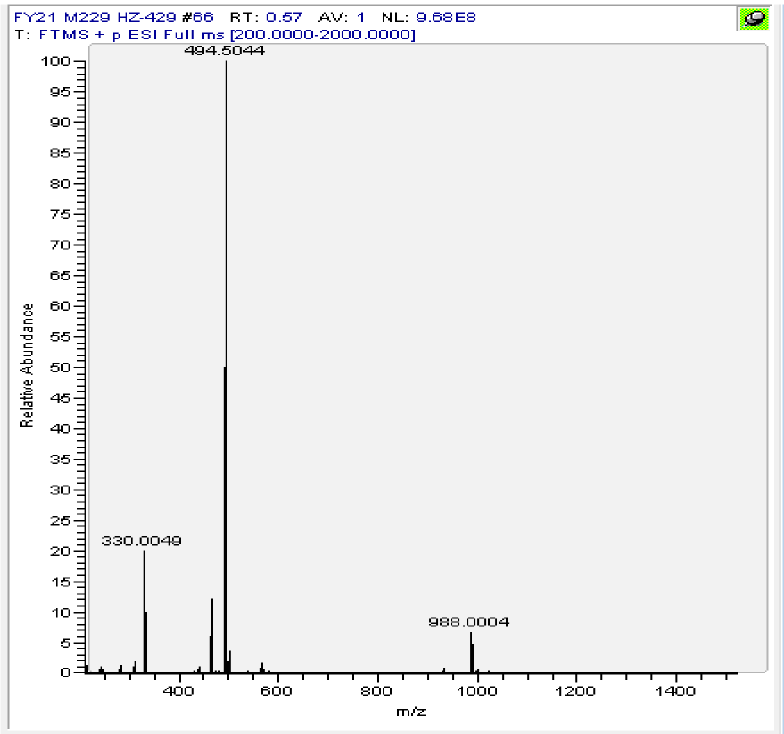
FY21 M229 HZ-429 #66 RT: 0.57 AV: 1 NL: 9.68E8
T: FTMS + p ESI Full ms [200.0000-2000.0000]
494.5044
330.0049
988.0004
Relative Abundance
m/z

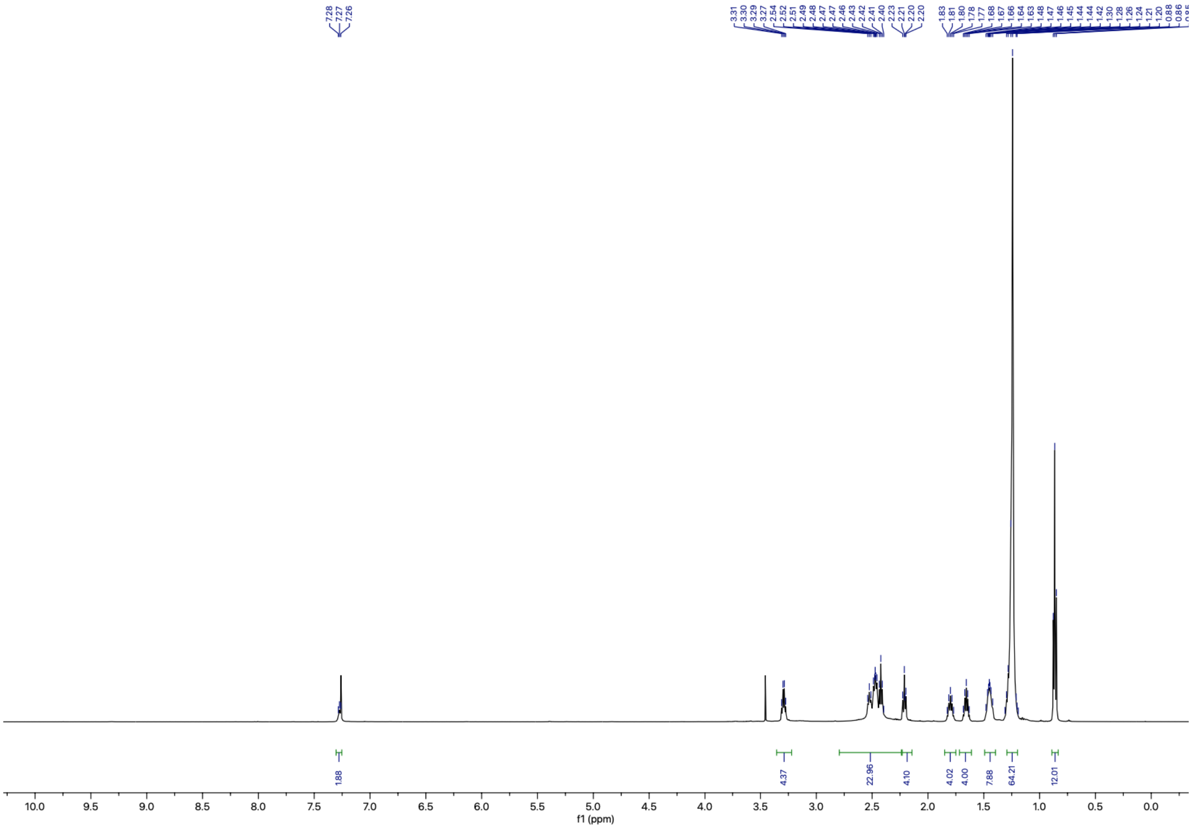
7.28
7.27
7.26
3.31
3.30
3.29
3.27
2.54
2.52
2.51
2.49
2.48
2.47
2.47
2.46
2.43
2.42
2.41
2.40
2.23
2.21
2.20
2.20
1.83
1.81
1.80
1.78
1.77
1.68
1.67
1.66
1.64
1.63
1.48
1.47
1.46
1.45
1.44
1.44
1.42
1.30
1.28
1.26
1.24
1.21
1.20
0.88
0.86
1.88
4.37
22.96
4.10
4.02
4.00
7.88
64.21
12.01
10.0
9.5
9.0
8.5
8.0
7.5
7.0
6.5
6.0
5.5
5.0
4.5
4.0
3.5
3.0
2.5
2.0
1.5
1.0
0.5
0.0
f1 (ppm)

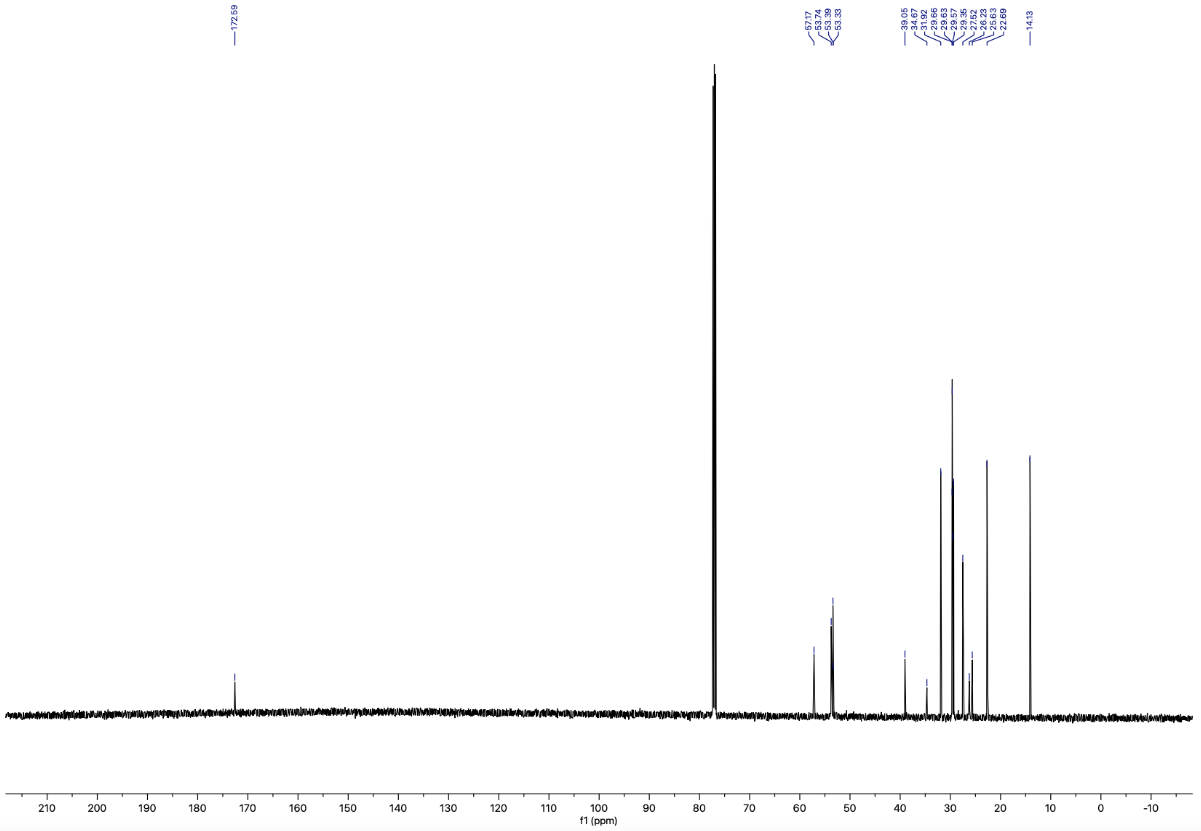
172.59
57.17
53.74
53.39
53.33
39.05
34.67
31.92
29.66
29.63
29.57
29.35
27.52
26.23
25.63
22.69
14.13
210
200
190
180
170
160
150
140
130
120
110
100
90
80
70
60
50
40
30
20
10
0
-10
f1 (ppm)

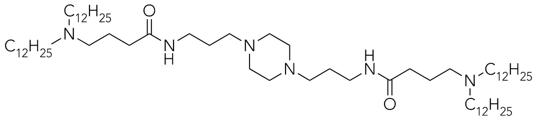

PPZ-B12

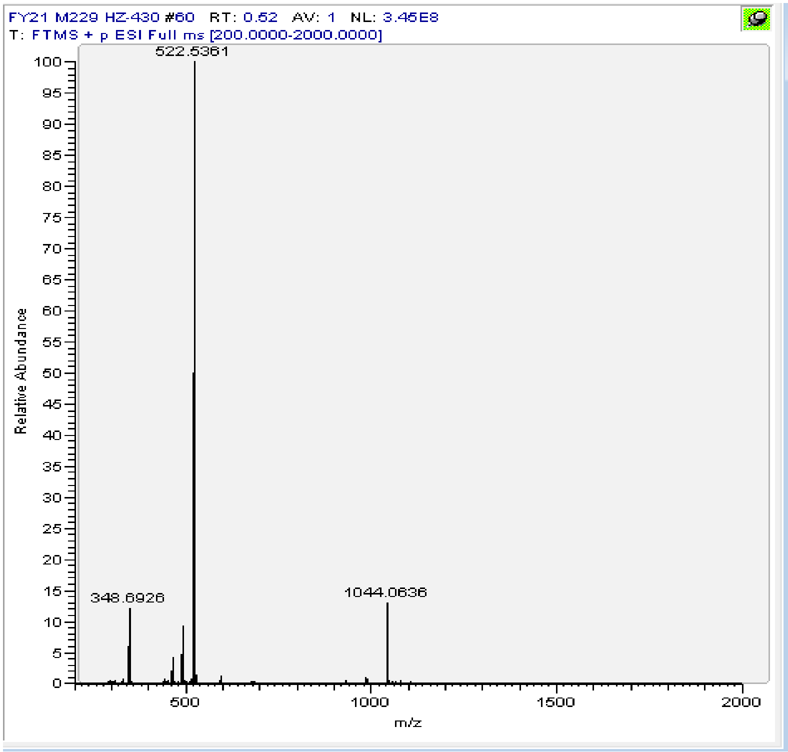

FY21 M229 HZ-430 #60 RT: 0.52 AV: 1 NL: 3.45E8
T: FTMS + p ESI Full ms [200.0000-2000.0000]
522.5361
348.6926
1044.0636
Relative Abundance
m/z

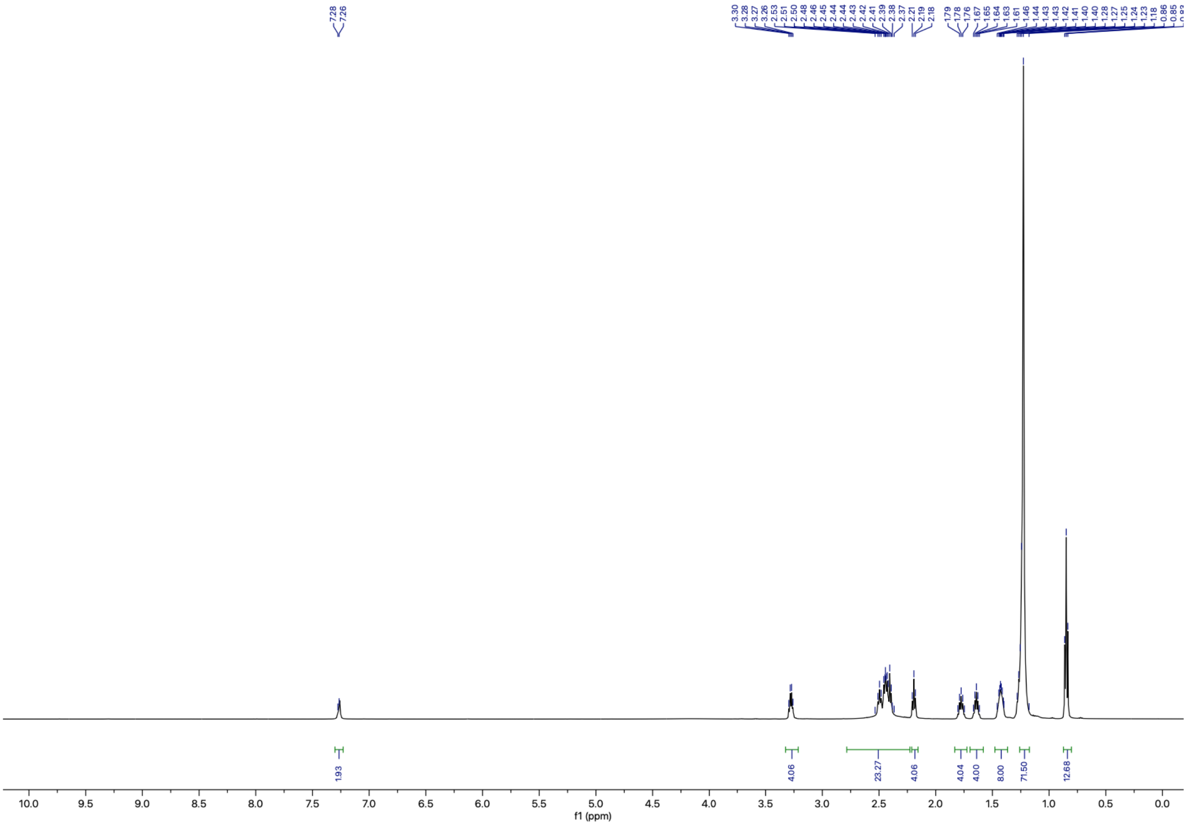

f1 (ppm)

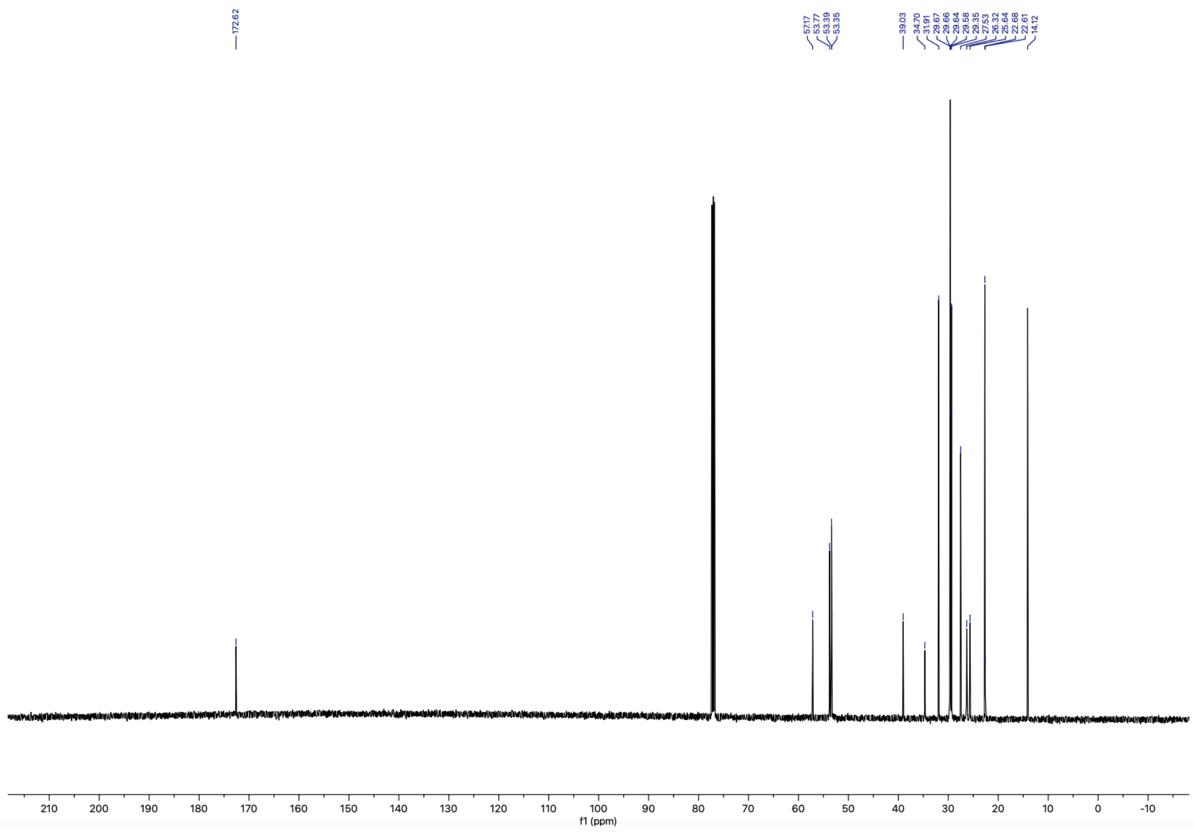
172.62
57.17
53.77
53.39
53.35
39.03
34.70
31.91
29.67
29.66
29.64
29.58
29.35
27.53
26.32
25.64
22.68
22.61
14.12
210 200 190 180 170 160 150 140 130 120 110 100 90 80 70 60 50 40 30 20 10 0 -10
f1 (ppm)

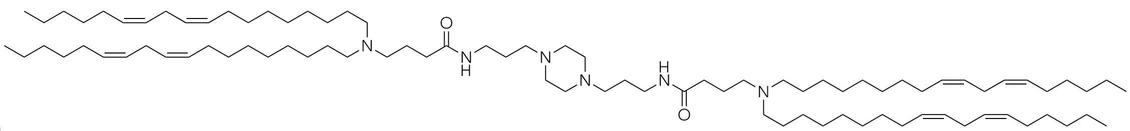
O
N
H
N
N
H
N
O
N
N

PPZ-B18-2Z

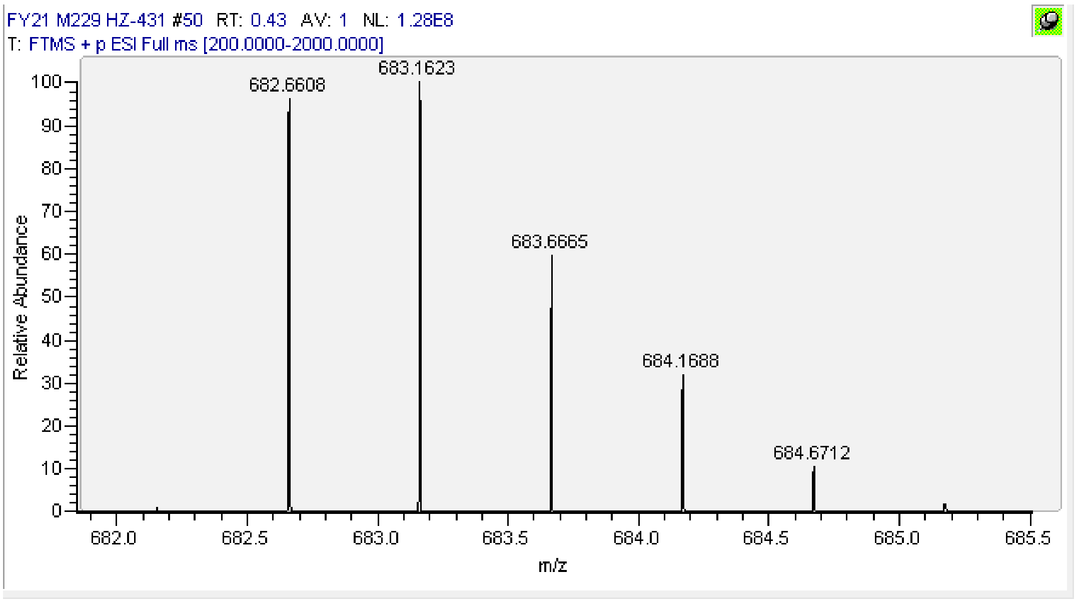
FY21 M229 HZ-431 #50 RT: 0.43 AV: 1 NL: 1.28E8
T: FTMS + p ESI Full ms [200.0000-2000.0000]
682.6608
683.1623
683.6665
684.1688
684.6712
Relative Abundance
100
90
80
70
60
50
40
30
20
10
0
682.0
682.5
683.0
683.5
684.0
684.5
685.0
685.5
m/z

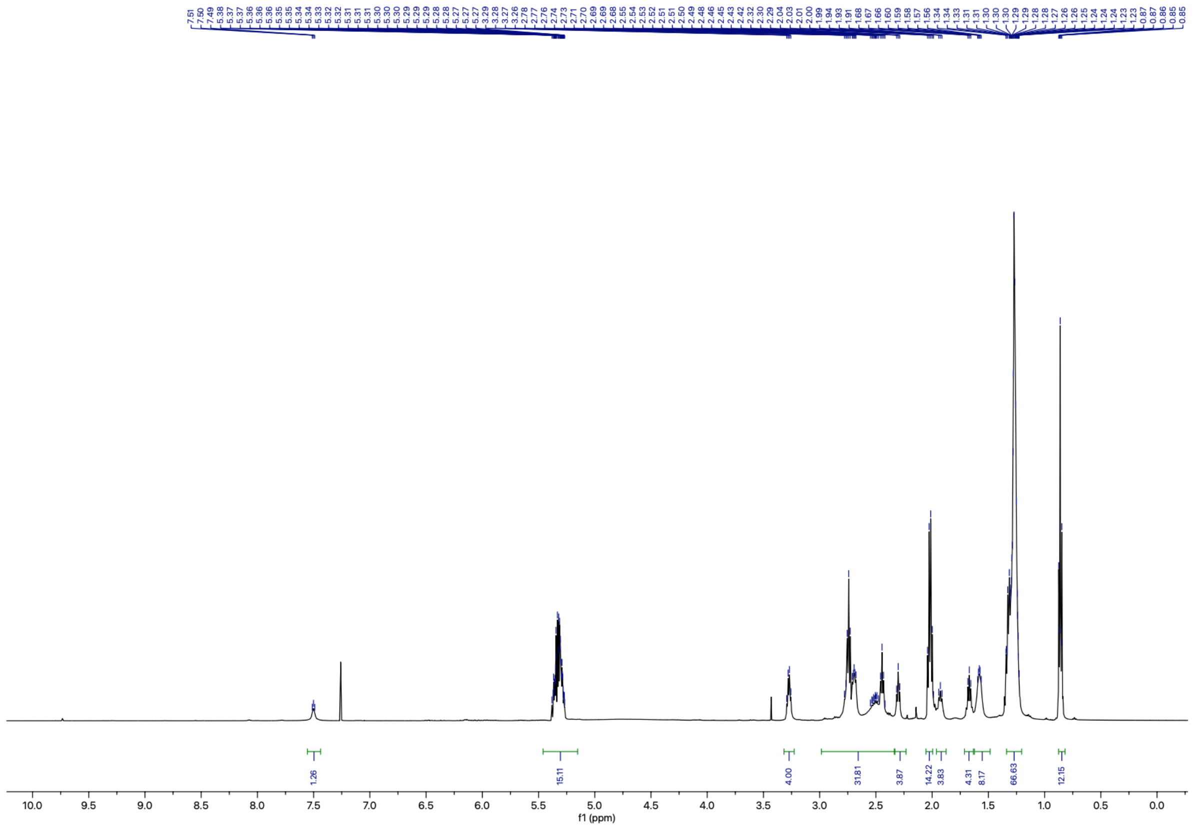
7.51 7.50 7.49 5.38 5.37 5.37 5.36 5.36 5.36 5.35 5.35 5.34 5.34 5.33 5.32 5.32 5.31 5.31 5.30 5.30 5.30 5.29 5.29 5.28 5.28 5.27 5.27 5.27 3.29 3.28 3.27 3.26 2.78 2.77 2.76 2.74 2.73 2.71 2.70 2.69 2.69 2.68 2.55 2.54 2.53 2.52 2.51 2.51 2.50 2.49 2.48 2.46 2.45 2.43 2.42 2.32 2.30 2.29 2.04 2.03 2.01 2.00 1.99 1.94 1.93 1.91 1.68 1.67 1.66 1.60 1.59 1.58 1.57 1.56 1.34 1.34 1.33 1.31 1.31 1.30 1.30 1.29 1.29 1.28 1.28 1.27 1.26 1.26 1.25 1.24 1.24 1.23 0.87 0.87 0.86 0.85 0.85
1.26
15.11
4.00
31.81
3.87
14.22
3.83
4.31
8.17
66.63
12.15
10.0 9.5 9.0 8.5 8.0 7.5 7.0 6.5 6.0 5.5 5.0 4.5 4.0 3.5 3.0 2.5 2.0 1.5 1.0 0.5 0.0
f1 (ppm)

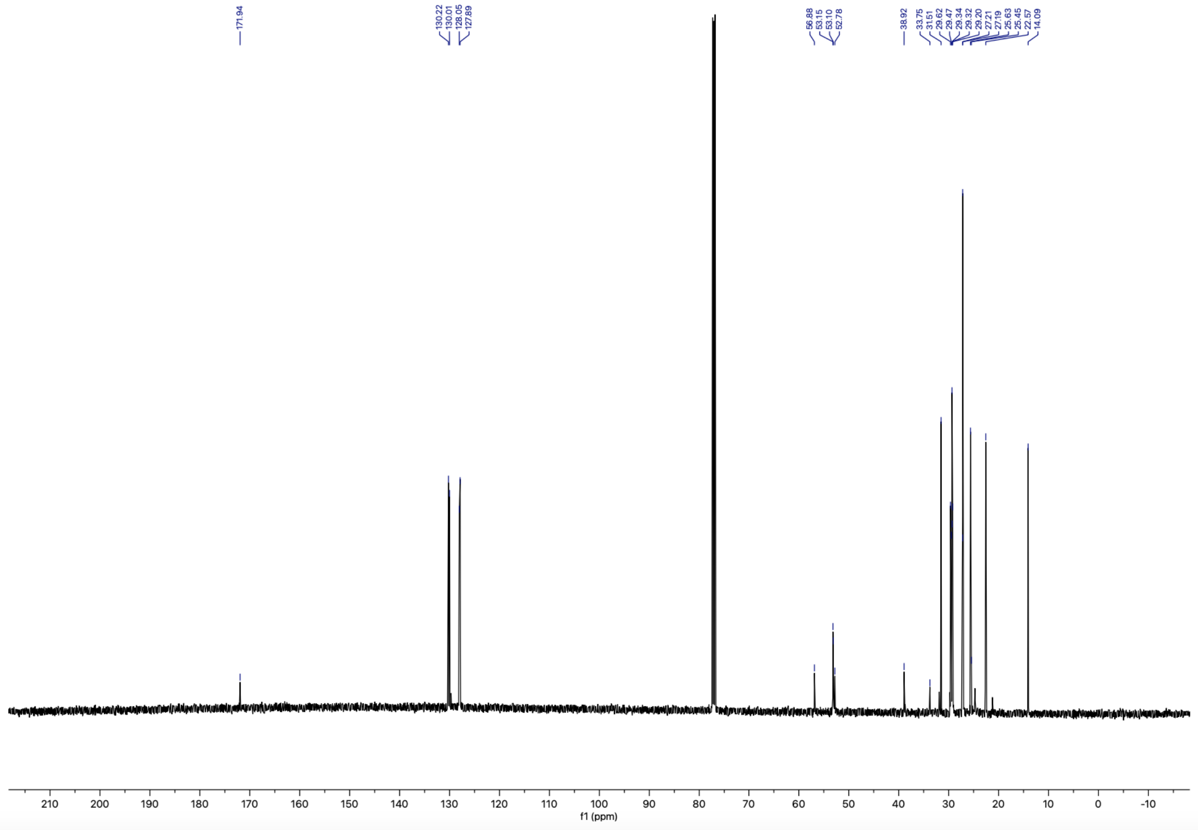
171.94
130.22 130.01 128.05 127.89
56.88 53.15 53.10 52.78
38.92 33.75 31.51 29.62 29.47 29.34 29.32 29.20 27.21 27.19 25.63 25.45 22.57 14.09
210 200 190 180 170 160 150 140 130 120 110 100 90 80 70 60 50 40 30 20 10 0 -10
f1 (ppm)
