# Supplementary material for: Piperazine-derived lipid nanoparticles deliver mRNA to immune cells in vivo
Source: Nat Commun. 2022 Aug 15;13:4766. doi: 10.1038/s41467-022-32281-5 (PMC9376583; doi:10.1038/s41467-022-32281-5)
Supplement: Supplementary file 2 — Reporting Summary [file 41467_2022_32281_MOESM2_ESM.pdf]

nature research

Corresponding author(s): James Dahlman

Last updated by author(s): Jul 6, 2022

Reporting Summary

Nature Research wishes to improve the reproducibility of the work that we publish. This form provides structure for consistency and transparency in reporting. For further information on Nature Research policies, see our Editorial Policies and the Editorial Policy Checklist.

Statistics

For all statistical analyses, confirm that the following items are present in the figure legend, table legend, main text, or Methods section.

n/a	Confirmed	
☐	☒	The exact sample size (n) for each experimental group/condition, given as a discrete number and unit of measurement
☐	☒	A statement on whether measurements were taken from distinct samples or whether the same sample was measured repeatedly
☐	☒	The statistical test(s) used AND whether they are one- or two-sided<br>*Only common tests should be described solely by name; describe more complex techniques in the Methods section.*
☒	☐	A description of all covariates tested
☒	☐	A description of any assumptions or corrections, such as tests of normality and adjustment for multiple comparisons
☐	☒	A full description of the statistical parameters including central tendency (e.g. means) or other basic estimates (e.g. regression coefficient) AND variation (e.g. standard deviation) or associated estimates of uncertainty (e.g. confidence intervals)
☐	☒	For null hypothesis testing, the test statistic (e.g. F, t, r) with confidence intervals, effect sizes, degrees of freedom and P value noted<br>*Give P values as exact values whenever suitable.*
☒	☐	For Bayesian analysis, information on the choice of priors and Markov chain Monte Carlo settings
☒	☐	For hierarchical and complex designs, identification of the appropriate level for tests and full reporting of outcomes
☒	☐	Estimates of effect sizes (e.g. Cohen's d, Pearson's r), indicating how they were calculated

Our web collection on statistics for biologists contains articles on many of the points above.

Software and code

Policy information about availability of computer code

Data collection	In vivo cell populations were analyzed and isolated using the BD FacsFusion in the Georgia Institute of Technology Cellular Analysis Core. Sequencing data was collected with an Illumina NextSeq High Throughput Flow Cell.
Data analysis	Data were analyzed in GraphPad Prism. The specifics of the data analyses are described in the text. Flow cytometry data was analyzed using FlowJo.

For manuscripts utilizing custom algorithms or software that are central to the research but not yet described in published literature, software must be made available to editors and reviewers. We strongly encourage code deposition in a community repository (e.g. GitHub). See the Nature Research guidelines for submitting code & software for further information.

Data

Policy information about availability of data

All manuscripts must include a data availability statement. This statement should provide the following information, where applicable:

- Accession codes, unique identifiers, or web links for publicly available datasets
- A list of figures that have associated raw data
- A description of any restrictions on data availability

All data are represented in the main figures or supplementary figures.

Field-specific reporting

Please select the one below that is the best fit for your research. If you are not sure, read the appropriate sections before making your selection.

- [x] Life sciences
- [ ] Behavioural & social sciences
- [ ] Ecological, evolutionary & environmental sciences

For a reference copy of the document with all sections, see nature.com/documents/nr-reporting-summary-flat.pdf

Life sciences study design

All studies must disclose on these points even when the disclosure is negative.

Sample size	For in vivo experiments: N = 2 for PBS negative control and 3 or 4 mice for experimental control. Those sample sizes were chosen to ensure the accuracy of the data and accurate statistics. The 2 mice per PBS control were chosen to limit the number of mice. No statistical method was used to predetermine sample size.
Data exclusions	No data were excluded from the analyses.
Replication	LNPs were confirmed at least three times independently and successfully.
Randomization	Mice were randomly selected, and no algorithm was used.
Blinding	The Investigators were not blinded to allocation during experiments and outcome assessment. Blinding investigators was not necessary to perform this research

Reporting for specific materials, systems and methods

We require information from authors about some types of materials, experimental systems and methods used in many studies. Here, indicate whether each material, system or method listed is relevant to your study. If you are not sure if a list item applies to your research, read the appropriate section before selecting a response.

Materials & experimental systems

n/a	Involved in the study	
	x	Antibodies
x		Eukaryotic cell lines
x		Palaeontology and archaeology
	x	Animals and other organisms
x		Human research participants
x		Clinical data
x		Dual use research of concern

Methods

n/a	Involved in the study	
x		ChIP-seq
	x	Flow cytometry
x		MRI-based neuroimaging

Antibodies

Antibodies used	The antibody clones used were the following: anti-CD31 (390, BioLegend), anti-CD45.2 (104, BioLegend), anti-CD68 (FA11, BioLegend), anti-CD11c (N418, Biolegend), and PE anti-mCD47 (miap301, BioLegend).
Validation	anti-CD31 (BioLegend, 390): "Anti-mouse CD31 clones 390 and MEC13.3 bind to their respective non-overlapping epitopes in IgD2 of CD31. CD31 is a 130-140 kD glycoprotein, also known as platelet endothelial cell adhesion molecule (PECAM-1) and EndoCAM". anti-CD45.2 (BioLegend, 104): "CD45.2 is an alloantigen of CD45, expressed by Ly5.2 bearing mouse strains (e.g., A, AKR, BALB/c, CBA/Ca, CBA/J, C3H/He, C57BL, C57BR, C57L, C58, DBA/1, DBA/2, NZB, SWR, 129). CD45, a member of the protein tyrosine phosphatase (PTP) family, is a 180-240 kD glycoprotein expressed on all hematopoietic cells except mature erythrocytes and platelets." anti-CD68 (FA11, BioLegend): "CD68 is an 85-115 kD member of the lysosomal-associated membrane protein (LAMP) family. It is expressed on tissue macrophages, Langerhans cells and at low levels on dendritic cells." anti-CD11c (N418, Biolegend): "CD11c is a 150 kD glycoprotein also known as αX integrin, CR4, and p150. It is primarily expressed on dendritic cells, NK cells, a subset of intestinal intraepithelial lymphocytes (IEL), and some activated T cells." anti-mCD47 (miap301, BioLegend): "CD47, also known as Integrin-Associated Protein (IAP), is a membrane protein of about 50 kD with an IgV-like extracelluar domain, a five membrane-spanning segment and a short terminal cytoplasmic region. It is widely expressed on many cell types and often associated with beta 3 integrins."

Animals and other organisms

Policy information about studies involving animals; ARRIVE guidelines recommended for reporting animal research

Laboratory animals	C57BL/6J (#000664) were purchased from The Jackson Laboratory. LSL-Tomato/Ai14 (#007914) were purchased from The Jackson Laboratory for breeding purposes. All mice were 6 to 8 weeks old at the time of the experiments. Both males and females were used. Light cycle of mice housing room are from 7 am to 7 pm. Housing room are kept at ~ 70 F with ~ 30% average humidity.
Wild animals	None
Field-collected samples	None
Ethics oversight	All animal experiments were performed in accordance with the Georgia Institute of Technology's IACUC (protocol number A100238).

Note that full information on the approval of the study protocol must also be provided in the manuscript.

Flow Cytometry

Plots

Confirm that:

- [x] The axis labels state the marker and fluorochrome used (e.g. CD4-FITC).
- [x] The axis scales are clearly visible. Include numbers along axes only for bottom left plot of group (a 'group' is an analysis of identical markers).
- [x] All plots are contour plots with outliers or pseudocolor plots.
- [x] A numerical value for number of cells or percentage (with statistics) is provided.

Methodology

Sample preparation	Cells were isolated 72 hours after injection with LNPs unless otherwise noted. Mice were perfused with 20 mL of 1X PBS through the right atrium. Liver tissues were finely minced, and then placed in a digestive enzyme solution with Collagenase Type I (Sigma Aldrich), Collagenase XI (Sigma Aldrich) and Hyaluronidase (Sigma Aldrich) at 37 ºC at 550 rpm for 45 minutes60, 61. Cell suspension was filtered through 70m mesh and red blood cells were lysed. Cells were stained to identify specific cell populations and sorted using the BD FacsFusion and BD Facs Aria IIIu cell sorters in the Georgia Institute of Technology Cellular Analysis Core.
Instrument	BD FacsFusion and BD Facs Aria IIlu
Software	The data were analyzed using FlowJo (BD Biosciences)
Cell population abundance	Greater than 1000.
Gating strategy	Cells were gated on FSC-A / SSC-A. Singlets were gated FSC-A / FSC-H. Cells types were gated by CD31 / CD45.2 then on CD68 / CD11c. PBS mice were used to gate tdTomato positive cells. See supporting figure 1 for additional information.

- [x] Tick this box to confirm that a figure exemplifying the gating strategy is provided in the Supplementary Information.
